# Modeling *PRPF31* retinitis pigmentosa using retinal pigment epithelium and organoids combined with gene augmentation rescue

**DOI:** 10.1038/s41536-022-00235-6

**Published:** 2022-08-16

**Authors:** Amélie Rodrigues, Amélie Slembrouck-Brec, Céline Nanteau, Angélique Terray, Yelyzaveta Tymoshenko, Yvrick Zagar, Sacha Reichman, Zhouhuan Xi, José-Alain Sahel, Stéphane Fouquet, Gael Orieux, Emeline F. Nandrot, Leah C. Byrne, Isabelle Audo, Jérôme E. Roger, Olivier Goureau

**Affiliations:** 1 Institut de la Vision, Sorbonne Université, INSERM, CNRS, 75012 Paris, France; 2 Paris-Saclay Institute of Neuroscience, CERTO-Retina France, CNRS, Université Paris-Saclay, 91400 Saclay, France; 3 Department of Ophthalmology, University of Pittsburgh School of Medicine, Pittsburgh, PA 15213 USA; 4 Centre Hospitalier National d’Ophtalmologie des Quinze-Vingts, Centre de référence maladies rares REFERET, INSERM-DHOS CIC 1423, 75012 Paris, France

**Keywords:** Retina, Retinal diseases

## Abstract

Mutations in the ubiquitously expressed *pre-mRNA processing factor* (*PRPF*) *31* gene, one of the most common causes of dominant form of Retinitis Pigmentosa (RP), lead to a retina-specific phenotype. It is uncertain which retinal cell types are affected and animal models do not clearly present the RP phenotype observed in *PRPF31* patients. Retinal organoids and retinal pigment epithelial (RPE) cells derived from human-induced pluripotent stem cells (iPSCs) provide potential opportunities for studying human *PRPF31*-related RP. We demonstrate here that RPE cells carrying *PRPF31* mutations present important morphological and functional changes and that *PRPF31*-mutated retinal organoids recapitulate the human RP phenotype, with a rod photoreceptor cell death followed by a loss of cones. The low level of *PRPF31* expression may explain the defective phenotypes of *PRPF31*-mutated RPE and photoreceptor cells, which were not observed in cells derived from asymptomatic patients or after correction of the pathogenic mutation by CRISPR/Cas9. Transcriptome profiles revealed differentially expressed and mis-spliced genes belonging to pathways in line with the observed defective phenotypes. The rescue of RPE and photoreceptor defective phenotypes by *PRPF31* gene augmentation provide the proof of concept for future therapeutic strategies.

## Introduction

Rod-cone dystrophies also called retinitis pigmentosa (RP) are the leading cause of blindness or visual impairment in the young adult population with an estimated prevalence of one in 3500 individuals and more than one million people affected worldwide^1,2^. RP forms a clinically and genetically heterogeneous group of inherited retinal disorders where rod photoreceptors and retinal pigment epithelial (RPE) cells are usually the first cells to degenerate, followed by secondary degeneration of cone photoreceptors. When the cone dysfunctions occur, progressive visual field constriction and loss of central vision may be observed. The disease can progress to very severe forms of visual impairment, even blindness, with no effective treatment so far. Mutations in over 80 genes have been identified and nearly 3100 mutations have been reported to date^2,3^. Mutations in the *pre-mRNA processing factors* (*PRPFs*), such as *PRPF3*, *PRPF4*, *PRPF6*, *PRPF8,* and *PRPF31*, are described as the second most common cause of autosomal dominant RP (adRP) after mutations in *RHODOPSIN*^2,3^. Among these factors, mutations in *PRPF31* are the most common with a prevalence ranging from 5 to 8% in adRP cohorts from various geographical origins^4–10^. Nearly 65 different mutations have been identified throughout the gene sequence, most commonly in exons 6–10^10–13^. The majority of reported *PRPF31* mutations are presumed loss-of-function variants including frameshift, splice site, nonsense, or large-scale insertions or deletions, which are predicted to lead to the complete loss of protein expression from the mutated allele. A consistent feature of *PRPF31*-associated RP is the incomplete penetrance with the presence of unaffected carriers that could be explained by an haploinsufficiency mechanism, in which the occurrence of the disease depends on higher or lower expressivity of the inherited normal allele^12,14–19^. *PRPF31* encodes a ubiquitously expressed splicing factor essential for the spliceosome activity^20^, and it remains unclear why mutations in this gene have an impact only on retinal cells. A high alternative splicing rate in the retina has been proposed to explain the enhanced sensitivity of these cells to spliceosome disturbance^21,22^. So far, the specific retinal cell type(s) affected by these mutations has not been clearly identified. Studies on *Prpf31*^*+/−*^ mice reported a morphological change in the aging RPE but not in the neural retina^23^, and showed a loss of RPE adhesion and a deficiency in phagocytosis of photoreceptor outer segments (POS)^24^. Therefore, these animal models are insufficient and there is a critical need of human models recapitulating the RP clinical phenotype to understand the mechanism underlying the disease, as well as the cellular origin of the disease. The development of human-induced pluripotent stem cells (hiPSCs) reprogrammed from somatic cells has brought new and powerful tools to model diseases, to study underlying cellular and molecular mechanisms, and to test new therapeutics. Many data have indicated that hiPSCs can be differentiated into the different retinal cell types^25–28^ affected in RP, including RPE cells and photoreceptors. Moreover, the advantage of patient iPSC-derived retinal cells is to recapitulate in vitro disease features caused by defined mutations within the diverse genetic background. Recent large-scale transcriptomic analysis using *PRPF31* patient-derived RPE cells and retinal organoids revealed disrupted alternative splicing of genes encoding pre-mRNA splicing proteins as well as genes involved in ciliogenesis and cellular adhesion^29^. The authors suggested that mis-splicing of these genes may be responsible for ultrastructural and functional abnormalities observed in these *PRPF31*-mutated RPE cells and photoreceptors^29,30^, in agreement with siRNA *PRPF31* knock-down approaches performed in human organotypic or cell line cultures^24,31–33^.

In this study, we present a substantial new resource of iPSCs from both clinically asymptomatic carriers and *PRPF31* patients affected by two different mutations, as well as isogenic controls, in order to gain further insight into *PRPF31*-associated RP and therapeutic response. Based on a robust retinal differentiation protocol established in our laboratory^34,35^, we clearly demonstrate a defective RPE phenotype and a photoreceptor cell death in 3D retinal organoids linked to the presence of *PRPF31* mutation, both of which can be prevented by *PRPF31* gene supplementation using the CRISPR/Cas9 strategy. We further demonstrate that the AAV-mediated gene therapy could effectively rescue the *PRPF31* mutation-induced photoreceptor cell death.

## Results

### Derivation and characterization of PRPF31 iPSCs

We derived fibroblasts from skin biopsies of two patients with genotype- and phenotype-confirmed RP who harbored mutations in *PRPF31*, either duplication in exon 8 (c.709_734dup) referred as Cys247X or deletion in exon 4 (c.269_273del) referred to as Tyr90CysfsX21, both causing premature termination of protein translation^10^. To establish control iPSC lines, a skin biopsy from an unaffected Cys247X family-related member (referred to as Control) was processed in parallel. Dermal fibroblasts were reprogrammed using non-integrative Sendai viruses expressing reprogramming factors *OCT4, KLF4, SOX2,* and *C-MYC*. Specific Sanger sequencing of generated iPSCs confirmed the presence of the mutation in the *PRPF31* gene for RP patients and homozygous normal reference sequence in the control (Supplementary Figs. 1a and 2a). Expanded hiPSC colonies displayed typical ES cell-like colony morphology, developed alkaline phosphatase activity (Supplementary Figs. 1b, c and 2b), and expressed pluripotency markers OCT4, SSEA4, NANOG, and TRA-1-60 (Supplementary Fig. 1c, d). These iPSCs did not exhibit any genomic abnormalities (Supplementary Figs. 1e and 2c), and showed complete silencing of the transgenes (Supplementary Fig. 1f). Finally, both patient-specific (Cys247X and Tyr90CysfsX21) and Control iPSCs were able to differentiate into the three embryonic germ cell layers evaluated by teratoma assay (Supplementary Fig. 1g) or in vitro differentiation (Supplementary Fig. 2e).

In order to generate an isogenic iPSC line, we repaired the c.709_734dup mutation (Cys247X) by CRISPR/Cas9 genome editing. A single-guide RNA (sgRNA) targeting the duplication in exon 8 and an ssODN template with the reference sequence were designed with homology arms on each side of the mutated region (Supplementary Fig. 3a). Around three hundred iPSC clones were selected and tested by PCR and eleven candidates identified by PCR (Supplementary Fig. 3b) were sequenced to confirm gene editing of *PRPF31*. To rule out any major chromosomal rearrangements that might have resulted from nucleofection or single-cell culture, we assessed the genomic integrity of the corrected iPSCs at mutational hotspots by the iCS-digital Pluri test, and selected one corrected iPSC clone that retained normal genomic stability (Supplementary Fig. 3c, d). This clone referred to as Cys247X-Isogenic (Cys247X-Iso) expressed pluripotency-associated markers OCT4, NANOG, SSEA4, and TRA-1-60 (Supplementary Fig. 3e). We also excluded potential off-target effects (Supplementary Data 1).

### RPE cells derived from clinically affected *PRPF31* patient cells have structural abnormalities and functional defects

**Fig. 1:**
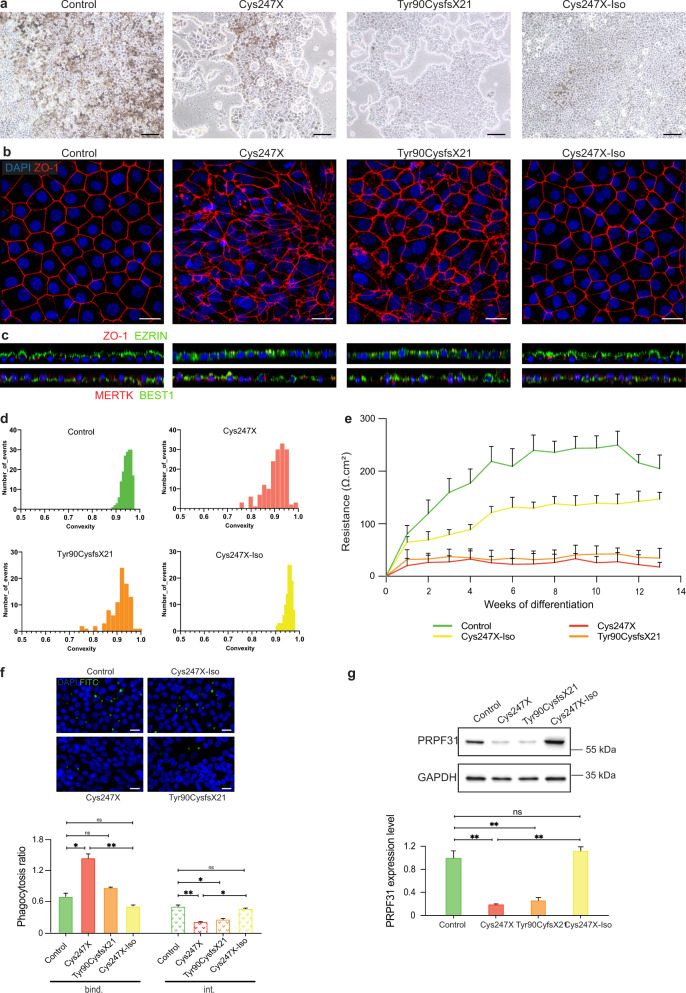
Characterization of RPE cells derived from *PRPF31*-mutated hiPSCs revealed morphological and functional abnormalities related to a lower level of PRPF31 protein expression. **a** Bright-field images of human iPSC-derived RPE cells from an unaffected family-related member (control), *PRPF31*-mutated (Cys247X and Tyr90CysfsX21), and isogenic controls (Cys247X-Iso) after 30 days of differentiation. Scale bar = 100 µm. **b** Immunostaining for tight-junction protein ZO-1. Nuclei were counterstained with DAPI. Scale bar = 10 µm. **c** Orthogonal projection of immunostaining for the apical protein Ezrin and the basolateral channel Bestrophin (BEST1). **d** Distribution of iRPE cells based on the convexity (ratio convex perimeter/perimeter) cell shape parameter from *N* = 3 independent differentiations. **e** Transepithelial resistance time course analysis of RPE cells between 1 and 13 weeks of culture on Transwell filters. **f** Representative images and quantitative analysis of iRPE cells phagocytic activity: ratios of FITC/DAPI fluorescence evaluated after 3-hour incubation with FITC-labeled POS. The fluorescence signal of bound particles (bind.) corresponds to the total fluorescence signal minus the fluorescence signal of internalized particles after quenching by trypan blue (int.). Values are mean ± SD, *n* = 9 samples from *N* = 3 independent differentiations. Statistical significance assessed using the Kruskal–Wallis test (*P < 0.05; **P < 0.01). Scale bar = 10 µm. **g** Representative image and quantitative analysis of western blots showing PRPF31 protein levels relative to GAPDH expression. Values are mean ± SD, *n* = 6 samples from *N* = 3 independent differentiations. Statistical significance assessed using Kruskal–Wallis test (**P < 0.01).

**Fig. 2:**
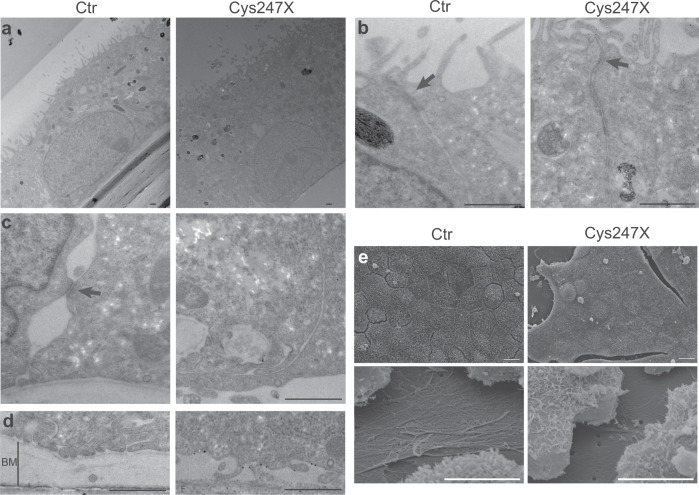
Electron microscopic analysis of control and *PRPF31*-mutated iRPE cells. **a**–**d** Transmission electronic microscopic images showed the presence of apical microvilli (**a**), basal nuclei (**a**), tight junctions (**b**, arrows) in both control (Ctr) and Cys247X iRPE cells, and the presence of desmosomes (**c**, arrow) and basal membranes (**d**, vertical line BM) only in Control iRPE cells. Scale bar = 1 µm. **e** Scanning electron microscopic images showed the presence of apical microvilli in both Control and Cys247X iRPE cells (upper panels) and the absence of basal membrane in Cys247X iRPE cells (lower panels). Scale bar = 10 µm.

IPSCs were differentiated into RPE cells using our previously reported retinal differentiation protocol^34^. For each experiment, RPE cells carrying *PRPF31* mutations (Cys247X and Tyr90CysfsX21) were grown for the same period of time and passaged in parallel with RPE cells derived from the unaffected family-related member (Control) and from the isogenic control (Cys247X-Iso). Control and Cys247X-Iso iPSC-derived RPE (iRPE) cells formed after 30 days a confluent pigmented cell monolayer with the classical honeycomb organization (Fig. 1a). However, iRPE cells from RP patients (Cys247X and Tyr90CysfX21) had less cell-to-cell contacts and seemed to be less adhesive leading to the appearance of many cell-free areas in the dish (Fig. 1a). Immunolabeling for tight-junction zonula occludens (ZO-1) showed an irregular staining in Cys247X and Tyr90CysfsX21 iRPE cells, whereas Control and Cys247X-Iso iRPE cells showed a typical polygonal morphology of RPE cells (Fig. 1b). *PRPF31*-mutated iRPE cells displayed an impaired polarization with the mis-localization of both apical protein Ezrin and the basolateral protein Bestrophin (BEST1**)** that did not appear restricted to their normal localization (Fig. 1c). Measurement of area and cell shape factors (convexity and geodesic diameter) of individual iRPE cells was performed from ZO-1 images after application of the Voronoi function and use of the MorpholibJ plugin (see Materials and Methods). The distribution of iRPE cells according to the morphometric criterion of convexity confirmed that Cys247X and Tyr90CysfsX21 iRPE cells show a much greater variety of shapes than Control and Cys247X-Iso iRPE cells (Fig. 1d). The same observation is made for the area and geodesic diameter of the cells (Supplementary Fig. 4), which confirms this greater irregularity of cell shape in iRPE cells carrying *PRPF31* mutation. Ultrastructural study by TEM and SEM revealed that both Control and Cys247X iRPE cells had basal nuclei, apical microvilli, melanin granules and tight junctions between adjoining cells near the apical part of the cells (Fig. 2a, b). However, no clear desmosomes and the absence of basal membranes were observed only in Cys247X iRPE cells (Fig. 2c–e). No significant transepithelial electrical resistance (TER) was recorded for Cys247X and Tyr90CysfsX21 iRPE cells during the 13 weeks of cultures (Fig. 1e), as expected due to the morphological appearance of the mutated RPE monolayer. To address a fundamental aspect of RPE cell function we assessed the ability of cells to carry out phagocytosis of FITC-labeled POS. A pronounced default was observed in Cys247X and Tyr90CysfsX21 iRPE cells, with a twofold decreased in POS internalization compared to Control and Cys247X-Iso iRPE cells (Fig. 1f). Western blot analysis showed a fivefold decrease in PRPF31 protein expression in Cys247X and Ty90CysfsX21 iRPE cells compared to Control and Cys247X-Iso iRPE cells (Fig. 1g), suggesting that a very low protein level of PRPF31 could explain the defective phenotype observed in *PRPF31*-mutated iRPE cells.

### PRPF31 haploinsufficiency leads to RPE phenotype

Haploinsufficiency has been suggested as a potential pathogenic mechanism to explain the incomplete penetrance of the *PRPF31*-associated phenotype^12,16,36^. It has been hypothesized that asymptomatic mutation carriers might express higher expression of the normal *PRPF31* allele depending on the nature of both mutant and normal inherited alleles^12^. To test this hypothesis, we used an iPSC line previously derived from fibroblasts of an asymptomatic patient harboring the *PRPF31* c.709_734dup mutation, referred to as Cys247X-Asymptomatic^37^. Furthermore, to investigate whether a higher level of *PRPF31* expression might be sufficient to reverse the phenotype, we engineered CRISPR/Cas9 knock-in (KI) iPSC lines into the PPP1R12C locus (referred to as AAVS1 site), in which a normal copy of the human *PRPF31* gene under the control of CAG ubiquitous promoter was inserted in both *PRPF31*-mutated iPSC lines Cys247X and Tyr90CysfsX21 (Supplementary Fig. 5). For each *PRPF31*-mutated iPSC line, we selected a puromycin-resistant clone carrying only one copy of the insert in the AAVS1 locus. These clones referred to as Cys247X-KI and Tyr90CysfsX21-KI showed normal morphology, absence of major chromosomal rearrangements, and expressed pluripotency-associated markers OCT4, NANOG, SSEA4, and TRA-1-60 (Supplementary Fig. 5).

**Fig. 3:**
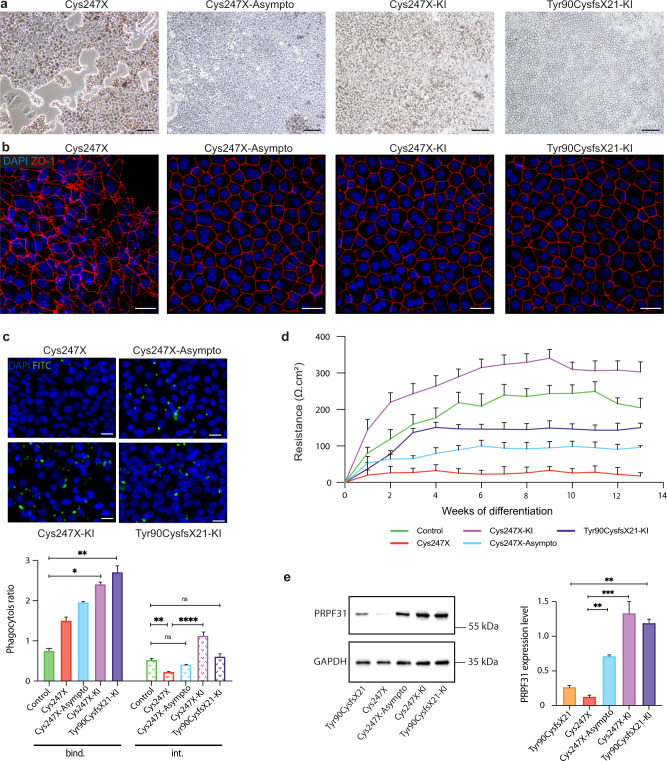
Defective RPE phenotype due to *PRPF31* mutations is not observed in an asymptomatic carrier and can be prevented by gene augmentation strategy. **a** Bright-field images of human iPSC-derived RPE cells from an affected patient carrying *PRPF31*-mutation (Cys247X), an unaffected patient carrying the same mutation (Cys247X-Asympto), an affected patient carrying one *PRPF31* mutation, and a supplementary WT copy of *PRPF31* in the AAVS1 locus (Cys247X-KI and Tyr90CysfsX21-KI), after 30 days of differentiation. Scale bar = 100 µm. **b** Immunostaining for the tight-junction protein ZO-1. Nuclei were counterstained with DAPI. Scale bar = 10 µm. **c** Representative images and quantitative analysis of RPE cell phagocytic activity: ratios of FITC/DAPI fluorescence evaluated after a 3-hour incubation with FITC-labeled POS. The fluorescence signal of bound particles (bind.) corresponds to the total fluorescence signal minus the fluorescence signal of internalized particles (int.) after quenching by trypan blue. Values are mean ± SD, *n* = 9 samples from *N* = 3 independent differentiations. Statistical significance assessed using the Kruskal–Wallis test (*P < 0.05; **P < 0.01; ****P < 0.0001). Scale bar = 10 µm. **d** Transepithelial resistance time course analysis of RPE cells between 1 and 13 weeks of cultured on Transwell filters. **e** Representative image and quantitative analysis of western blots showing PRPF31 protein levels relative to GAPDH expression. Values are mean ± SD, *n* = 6 samples from *N* = 3 independent differentiations. Statistical significance assessed using Kruskal–Wallis test (**P < 0.01; ***P < 0.001).

Both Cys247X-Asymptomatic (Cys247X-Asympto), Cys247X-KI, and Tyr90CysfsX21-KI iPSCs subjected to RPE cell differentiation formed a confluent cell monolayer displaying the classical RPE morphology (Fig. 3a) with clear cell membrane tight junctions identified by ZO-1 immunolabeling (Fig. 3b). Analysis of the distribution of iRPE cell population according to the three morphometric criteria (area, convexity, and geodesic diameter) revealed that Cys247X-Asympto, Cys247X-KI, and Tyr90CysfsX21-KI iRPE cells have a similar distribution to that observed for Control and Cys247X-Iso iRPE cells (Supplementary Fig. 4). POS phagocytosis assay showed similar internalization of POS in Cys247X-Asympto iRPE as for Control cells. Both Cys247X-KI and Tyr90CysfsX21-KI iRPE cells presented normal POS internalization when compared to their related *PRPF31*-mutated cells, with also a significant improvement in POS binding (Fig. 3c). Recurrent measurements taken over three months showed a significant increase in TER for the Cys247X-Asympto iRPE cells (~150 ohms/cm^2^) after 8 weeks of differentiation, in contrast to Cys247X iRPE cells that presented no significant TER (Fig. 3d). A TER value of the same order of magnitude as the one observed in Control iRPE cells can be recorded in *PRPF31* knock-in (Cys247X-KI and Tyr90CysfsX21) iRPE cells (Fig. 3d), suggesting the requirement of a sufficient threshold of PRPF31 expression to maintain RPE functionality. Indeed, western blot analysis confirmed a significantly higher level of PRPF31 protein expression in Cys247X-Asympto and in both Cys247X-KI and Tyr90CysfsX21-KI iRPE cells compared to Cys247X and Tyr90CysfsX21 iRPE cells (Fig. 3e).

### Progressive photoreceptor degeneration in retinal organoids derived from clinically affected PRPF31 patient cells

**Fig. 4:**
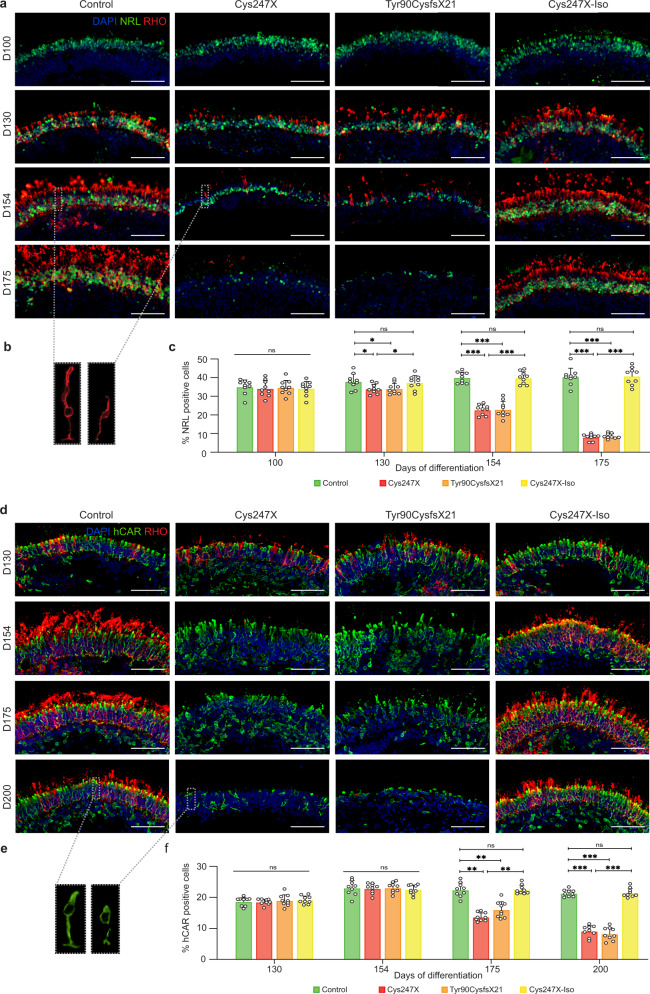
Mutations in *PRPF31* lead to a successive rod and cone photoreceptor degeneration in hiPSC-derived retinal organoids. **a** Immunofluorescence staining of retinal organoid cryosections derived from unaffected family-related member (Control), *PRPF31*-mutated (Cys247X and Tyr90CysfsX21) and isogenic control (Cys247X-Iso) hiPSCs after 100, 130, 154, and 175 days of differentiation (D100, D130, D154, and D175) using rod photoreceptor markers NRL and Rhodopsin (RHO). Nuclei were counterstained with DAPI. Scale bar = 100 µm. **b** Higher magnification of one representative rod cell stained with the RHO marker of control (left) and *PRPF31*-mutated (right) retinal organoids at D154. **c** Quantification of the percentage of NRL-positive cells in retinal organoids after different differentiation times. Each data point represents counts of NRL-positive cells relative to DAPI-positive cells performed on one-quarter of a representative cross-section of one organoid. Values are mean ± SD for each time point, *n* = 9 independent retinal organoids from *N* ≥ 3 independent differentiations. Statistical significance assessed using the Kruskal–Wallis test (*P < 0.05; ***P < 0.001). **d** Immunofluorescence staining of cryosections from retinal organoids at D130, D154, D175, and D200 using rod (RHO) and cone (human cone arrestin: hCAR) markers. Nuclei were counterstained with DAPI. Scale bar = 100 µm. **e** Higher magnification of one representative cone cell stained with hCAR marker of control (left) and *PRPF31*-mutated (right) retinal organoids at D200. **f** Quantification of the percentage of hCAR-positive cells in retinal organoids after different differentiation times. Each data point represents counts of hCAR-positive cells relative to DAPI-positive cells performed on one-quarter of a representative cross-section of one organoid. Values are mean ± SD, for each time point *n* = 9 independent retinal organoids from *N* ≥ 3 independent differentiations. Statistical significance assessed using the Kruskal–Wallis test (**P < 0.01; ***P < 0.001).

**Fig. 5:**
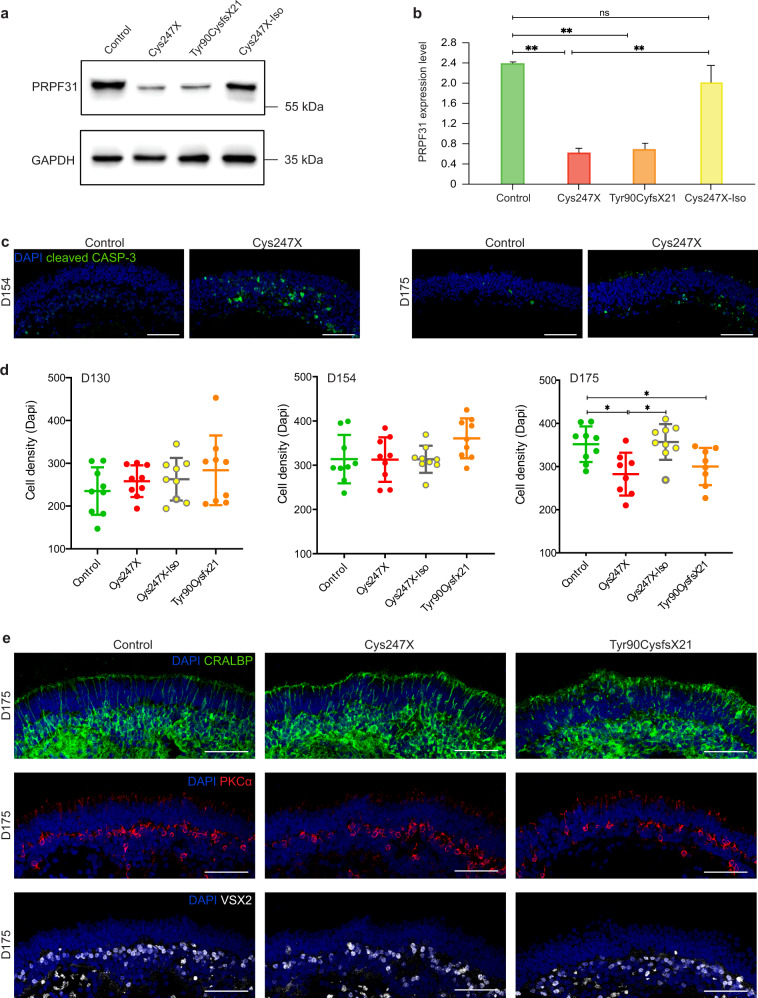
*PRPF31*-related retinal degeneration is correlated with lower levels of PRPF31 protein expression and does not affect bipolar or Müller glial cells. **a**, **b** Representative images and quantitative analysis of western blots showing PRPF31 protein levels relative to GAPDH expression in D130 retinal organoids. Values are mean ± SD, *n* = 6 samples from *N* = 3 independent differentiations. Statistical significance assessed using the Kruskal–Wallis test (**P < 0.01). **c** Immunofluorescence staining of cryosections from control and *PRPF31*-mutated (Cys247X) retinal organoids after 154 days of differentiation (D154) using a marker for the active form of the apoptotic factor Caspase-3 (cleaved CASP-3). Nuclei were counterstained with DAPI. Scale bar = 100 µm. **d** Quantification of the cell density of presumptive ONL in retinal organoids after different differentiation times. Each data point represents counts of DAPI-positive cells performed on one-quarter of a representative cross-section of one organoid. Values are mean ± SD, for each time, independent retinal organoids from *N* ≥ 3 independent differentiations. Statistical significance assessed using the One-way ANOVA test (*P < 0.05). **e** Immunolabeling of cryosections from D175 retinal organoids using markers for Müller glial (CRALBP) and bipolar (PKCα, VSX2) cells. Nuclei were counterstained with DAPI. Scale bar = 100 µm.

Different iPSCs carrying *PRPF31* mutations (Cys247X and Tyr90cysfsX21), related Control (unaffected related family member), and isogenic control (Cys247X-Iso) were differentiated into pseudo-laminated retinal organoids using our recently optimized retinal differentiation protocol^35^ in order to follow photoreceptor cell maturation and/or cell death between 100 and 200 days of culture. In early retinal organoids, no significant difference was observed for the rod-specific photoreceptor marker neural retina-specific leucine zipper (NRL) after 100 days of culture (Fig. 4a) nor the cone-specific marker human cone arrestin (hCAR) labeling after 130 days of culture (Fig. 4d), suggesting that *PRPF31* mutations did not affect rod and cone photoreceptor differentiation. However, a striking decrease of NRL staining occurred after D130 in Cys247X and Tyr90CysfsX21 organoids (Fig. 4a), with a decrease in the number of NRL-positive rods reaching <10% in these structures at D175 (Fig. 4c). A similar degenerative profile of rods was observed with rhodopsin staining (Fig. 4a, d), showing also a severe alteration of rod morphology in particular in outer segments and synaptic terminal regions in the presence of *PRPF31* mutations (Fig. 4b). A significant decrease in hCAR-positive cones was observed by immunostaining at D175 and D200 in Cys247X and Tyr90CysfsX21 organoids compared to organoids derived from Control and isogenic corrected iPSCs (Fig. 4d, f). The relatively late morphological changes in cone photoreceptors (Fig. 4e) and their later cell death suggest that cones may undergo a degenerative process following rod cell death as observed in RP patients. Importantly, the similar profile of rod and cone maturation and the absence of photoreceptor cell degeneration in Cys247X-Iso and Control organoids confirmed that the RP phenotype observed in *PRPF31*-mutated organoids is solely due to the presence of the *PRPF31* mutation. We performed western blot analysis at D130, when rod cell number started to decrease but before the photoreceptor degeneration became too severe, to compare levels of PRPF31 expression between Control and *PRPF31*-mutated organoids. We showed a strong decrease in PRPF31 protein expression in Cys247X and Ty90CysfsX21 organoids compared to Control and Cys247X-Iso organoids (Fig. 5a, b), suggesting that the typical RP phenotype observed in *PRPF31*-mutated organoids is related to a significantly lower level of PRPF31 proteins. Positive staining for the cleaved caspase-3 (Cleaved CASP-3) confirmed the presence of apoptotic cells through the outer nuclear layer-like (ONL) in *PRPF31*-mutated organoids (Cys247X) (Fig. 5c). Assessment of cell density in the presumptive ONL between D130 and D175, showed that D175 retinal organoids from Cys247X and Tyr90CysfsX21 iPSCs had a significant lower ONL cell density compared to organoids derived from Control and isogenic corrected (Cys247X-Iso) iPSCs (Fig. 5d), reflecting photoreceptor cell death. Regarding the other cell types present in retinal organoids, immunolabeling with bipolar cell (VSX2, PKCα) and Müller glial cell (CRALBP) markers revealed no differences between *PRPF31*-mutated and Control organoids (Fig. 5e), thus confirming that the retinal degeneration due to *PRPF31* mutations is restricted to photoreceptors.

### RP-like phenotype observed in retinal organoids is due to PRPF31 haploinsufficiency

**Fig. 6:**
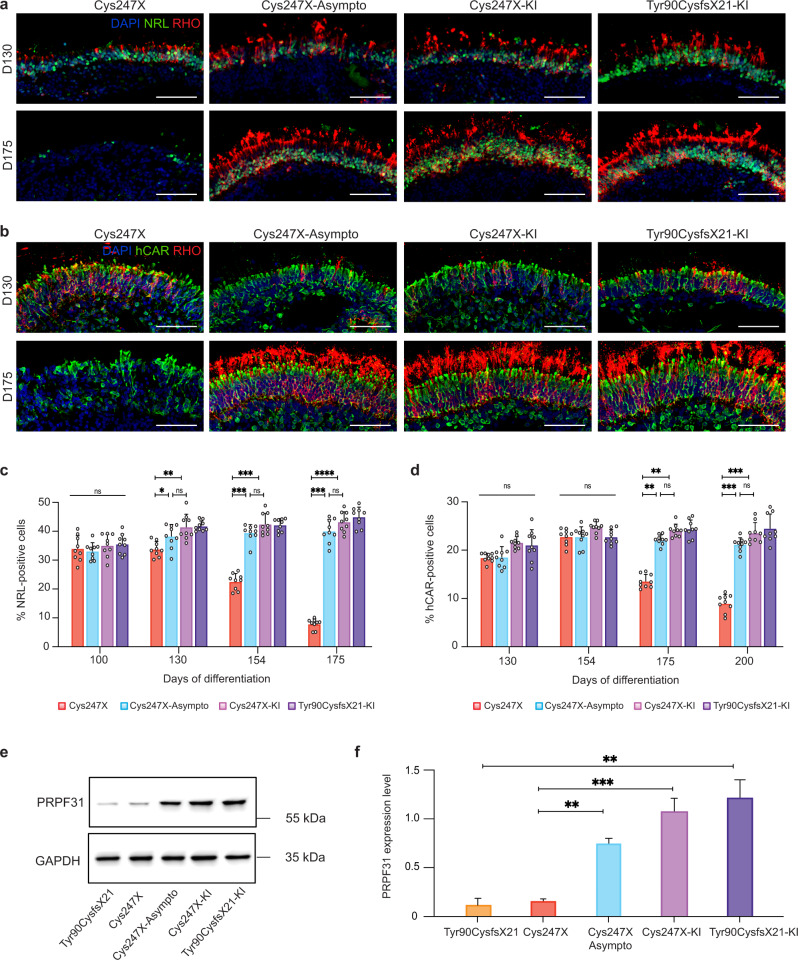
Photoreceptor degeneration is not observed in retinal organoids derived from an asymptomatic carrier and can be prevented by gene augmentation strategy. **a** Immunofluorescence staining of retinal organoid cryosections derived from an affected patient carrying mutation (Cys247X), an unaffected patient carrying the same mutation (Cys247X-Asympto), and *PRPF31*-KI (Cys247X-KI and Tyr90CysfsX21-KI) iPSCs after 130 and 175 days of differentiation (D130, and D175) using rod photoreceptor markers NRL and Rhodopsin (RHO). Nuclei were counterstained with DAPI. Scale bar = 100 µm. **b** Immunofluorescence staining of retinal organoid cryosections at D130 and D175 using markers for rod (RHO) and cone (hCAR) photoreceptors. Nuclei were counterstained with DAPI. Scale bar = 100 µm. **c** Quantification of the percentage of NRL-positive cells in retinal organoids after different differentiation times (D100, D130, D154, and D175). **d** Quantification of the percentage of hCAR-positive cells in retinal organoids after different differentiation times (D130, D154, D175, and D200). Each data point represents counts of NRL or hCAR-positive cells relative to DAPI-positive cells performed on one-quarter of a representative cross-section of one organoid. Values are mean ± SD, for each time point *n* = 9 independent retinal organoids from *N* ≥ 3 independent differentiations. Statistical significance assessed using the Kruskal–Wallis test (*P < 0.05; **P < 0.01; ***P < 0.001; ****P < 0.0001). **e** Representative image and **f** quantitative analysis of western blots showing PRPF31 protein levels relative to GAPDH expression in retinal organoids at D130. Values are mean ± SD, *n* = 6 samples from *N* = 3 independent differentiations. Statistical significance assessed using the Kruskal–Wallis test (**P < 0.01; ***P < 0.001).

As for previously reported RPE phenotypes, we have investigated if a haploinsufficiency mechanism could explain *PRPF31*-related photoreceptor degeneration using Cys247X-Asympto and PRPF31-KI iPSCs. Early- and late-stage retinal organoids were immunostained with early and mature rod markers NRL and RHO, respectively, and with the cone marker hCAR (Fig. 6a, b). From D130 to D175, when rod degeneration is observed in Cys247X organoids, the percentage of NRL-positive rods was significantly higher in Cys247X-Asympto, Cys247X-KI, and Tyr90CysfsX21-KI than in Cys247X organoids (Fig. 6c). The same observation was made for cone photoreceptors at D200, where retinal organoids derived from the unaffected carrier of *PRPF31* mutation (Cys247X-Asympto) or with a supplementary copy of wild-type *PRPF31* (Cys247X-KI and Tyr90CysfsX21-KI) displayed around 50% more hCAR-positive cells (Fig. 6d). The absence of this RP phenotype in retinal organoids derived from Cys247X-Asympto and *PRPF31*-KI (Cys247X-KI and Tyr90CysfsX21-KI) iPSCs was linked to a significant higher level of PRPF31 protein expression. Indeed, western blot analysis demonstrated a 5–7-fold increase in PRPF31 expression between Cys247X and Cy247X-Asympto, Cys247X and Cys247X-KI, and Tyr90CysfsX21 and Tyr90CysfsX21-KI D130 organoids (Fig. 6e, f).

### AAV-driven PRPF31 gene augmentation improves photoreceptor cell survival

**Fig. 7:**
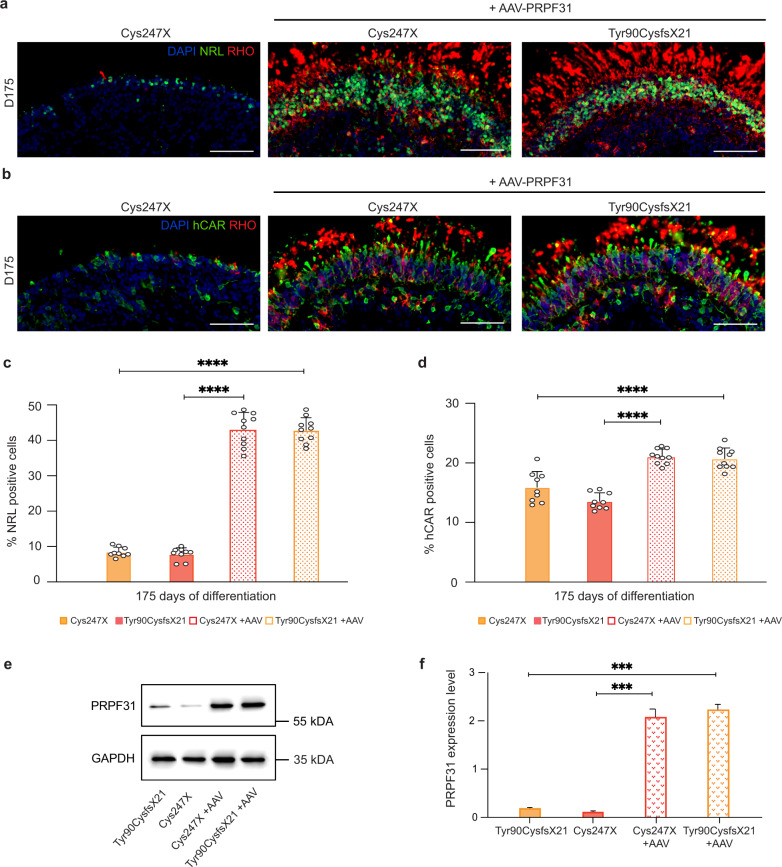
Photoreceptor degeneration phenotype can be rescued by AAV-driven *PRPF31* overexpression. **a**, **b** Immunofluorescence staining of cryosections of *PRPF31*-mutated (Cys247X) retinal organoids and *PRPF31*-mutated retinal organoids transduced with PRPF31-AAV (Cys247X + AAV-*PRPF31*) at D175 using markers for rod (NRL and RHO) and cone (hCAR) photoreceptors. Nuclei were counterstained with DAPI. Scale bar = 100 µm. **c**, **d** Quantification of the percentage of NRL or hCAR-positive cells in D175 retinal organoids. Each data point represents manual counts of NRL or hCAR-positive cells relative to DAPI-positive cells performed on one-quarter of a representative cross-section of one organoid. Values are mean ± SD, for each time point *n* ≥ 9 independent retinal organoids from *N* ≥ 3 independent differentiations. Statistical significance assessed using the Kruskal–Wallis test (****P < 0.0001). **e** Representative image and **f** quantitative analysis of western blots showing PRPF31 protein levels relative to GAPDH expression in retinal organoids at D130. Values are mean ± SD, *n* = 6 samples from *N* = 3 independent experiments. Statistical significance assessed using the Kruskal–Wallis test (***P < 0.001).

From the observations obtained with the KI approach by CRISPR/Cas9, we tested whether AAV-mediated *PRPF31* gene augmentation can rescue this degenerative phenotype in retinal organoids. The variant AAV7m8 was chosen for its high transduction efficiency and long-lasting expression after infection without compromising cell viability when applied to iPSC-derived retinal organoids^38,39^. The optimal balance between high efficiency and viability was determined by AAV2-7m8-GFP (AAV-GFP) transduction of organoids at D85, prior photoreceptor degeneration. The clear GFP staining detected on D175 organoid cryosections confirmed that this approach was particularly efficient to transduce the ONL-like structure from retinal organoids (Supplementary Fig. 6) where photoreceptors are mainly located. In addition, no toxicity effects or morphological changes were observed. Under this experimental condition, transduction of AAV2-7m8-*PRPF31* vector (AAV-*PRPF31*) on organoids carrying *PRPF31* mutations (Cys247X or Tyr90CysfsX21) restored both NRL, Rhodopsin, and hCAR immunostaining in D175 organoids (Fig. 7a, b). Cell quantification demonstrated that AAV-PRPF31-transduced organoids displayed ~40% of NRL-positive rods and 20% of hCAR-positive cones at D175 (Fig. 7c, d), consistent with values previously determined in Control organoids. Western blot analysis confirmed that after AAV treatment, PRPF31 expression increased 10- to 15-fold compared to endogenous PRPF31 expression (Fig. 7e, f). These results demonstrated that increasing PRPF31 expression levels directly in retinal organoids is efficient and sufficient to prevent photoreceptor degeneration.

### Whole transcriptome analysis from iRPE cells reveals altered expression of genes related to cell adhesion and extracellular matrix

**Fig. 8:**
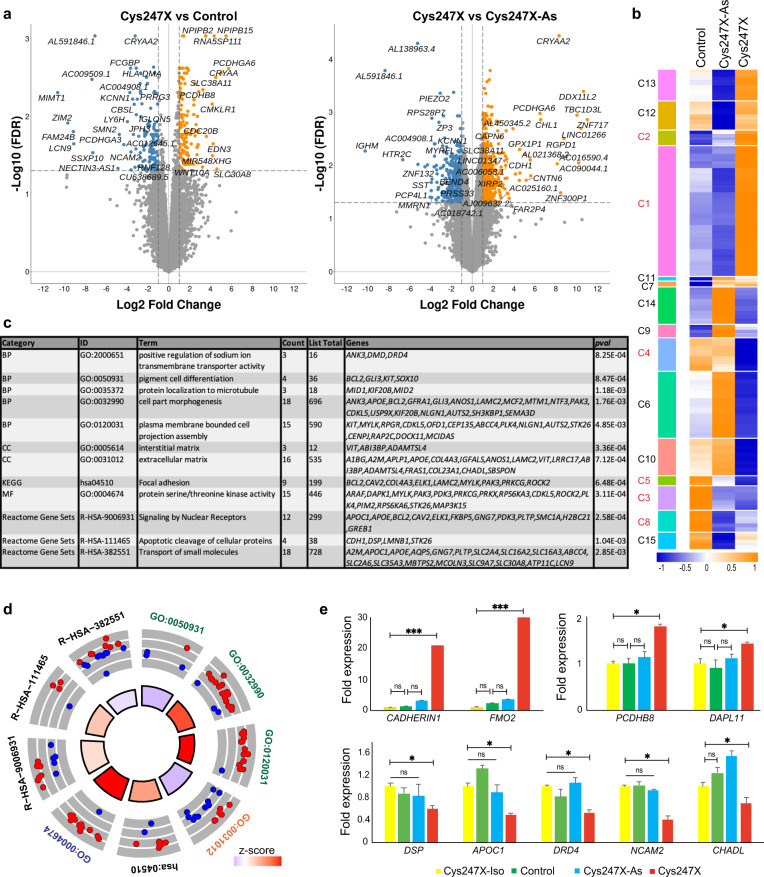
Identification of genes and pathways deregulated in hiPSC-derived RPE cells from *PRPF31* patients using whole transcriptome analysis. **a** Volcano plots of differentially expressed genes for Cys247X *vs* Control (left panel) and Cys247X *vs* Cys247X-As (right panel). Difference in gene expression (FC) is plotted on the x-axis (log2 scale), and False Discovery Rate (FDR) adjusted significance is plotted on the y-axis (log10 scale). Genes significantly up- or downregulated are indicated in orange and blue, respectively. **b** Hierarchical clustering analysis of the DEGs between all groups with genes up- or downregulated by a factor ≥2 with FDR ≤ 0.05 between Control, Cys247X-As and Cys247X patient-derived iRPE cells. The *z* score was derived from the average of the triplicates for each experimental group. Blue: low expression; orange: high expression. The left column represents the 15 clusters, and selected clusters used for further pathway analysis are in red. **c** Table of the over-represented GO pathways of interest identified with Metascape using the DEGs from the selected clusters. The different categories of pathways are BP, Biological Process; CC, Cell Compartment; KEGG, Kyoto Encyclopedia of Genes and Genomes; MF, Molecular Function. **d** Circular visualization of selected GO enriched pathways. Down- (blue dots) and up-regulated genes (red dots) within each GO pathway are plotted based on logFC. *z* score bars indicate if an entire biological process is more likely to be increased or decreased based on the genes it comprises. Green, Biological Process; orange, Cell Compartment; blue, Molecular Function. **e** Differential expression analysis by RT-qPCR of *CADHERIN1*, *FMO2*, *PCDHB8*, and *DAPL11* (upper panel) and of *DSP*, *APOC1*, *DRD4*, *NCAM2,* and *CHADL* (lower panel) in Cys247X-Iso, Control, Cys247X-As and Cys247X iRPE cells. Fold expression is relative to Cys247X-Iso. Values are mean ± SD, *n* = 3 samples of 1 × 10^6^ iRPE cells from *N* = 3 independent differentiations. Data were normalized against the geometric average ΔCt of the *18* *S* housekeeping gene. Statistical significance assessed using the Kruskal–Wallis test (*P < 0.05; ***P < 0.001).

In order to assess the transcriptional changes associated with the defective RPE phenotype observed in hiRPE cells carrying the *PRPF31* mutation, we performed whole transcriptome sequencing using biological triplicates from Control, Cys247X-Asympto (Cys247X-As) and Cys247X iRPE cells. Analysis of the expression level of well-known RPE-specific genes first confirmed the identity of iRPE cells (Supplementary Fig. 7). Filtering of the differentially expressed genes (DEGs) with a fold change (FC) greater or <2, an FDR (False Discovery Rate) lower than 0.05 and a minimum expression of five TPM led to the identification of 190 DEGs for Cys247X *vs* Control and 545 DEGs for Cys247X vs Cys247X-As (Fig. 8a). Hierarchical clustering based on z score allowed the selection of 6 clusters where genes are differentially expressed between Cys247X and both Control and Cys247X-As RPE cells (Fig. 8b). These 6 clusters encompassed 337 DEGs including several adhesion molecules such as seven members of the protocadherin/cadherin family, which were found among the most differentially expressed in iRPE cells carrying a *PRPF31* mutation (Supplementary Fig. 8 and Supplementary Data 2). Pathway enrichment analysis of the selected DEGs confirmed a large deregulation of extracellular matrix genes and genes related to cell adhesion, such as *Claudin 3* and *19* (Supplementary Fig. 7). These transmembrane proteins have been identified as major components of RPE tight junctions^40,41^, and the defective adhesion phenotype observed in *PRPF31*-mutated iRPE cells could be related to their downregulated expression. Other identified pathways related to transport, pigment cell differentiation, and cytoskeleton were also in line with our cellular observations (Fig. 8c). Circular visualization based on the calculated *z* score indicated that most of enriched pathways included up-regulated DEGs in Cys247X iRPE cells, including some genes belonging to apoptotic cleavage of cellular proteins (Fig. 8d). Genes related to the extracellular matrix were mostly downregulated (11/16). Based on our whole transcriptome analysis, the differential expression on few selected genes belonging to the most relevant enrichment pathways, were confirmed by RT-qPCR (Fig. 8e). Importantly, expression of these 9 deregulated genes was back to control levels in the Cys247X-Iso iRPE cells confirming the deregulation of their expression is due to the presence of *PRPF31* mutations. Interestingly, expression of these genes in iRPE cells from unaffected carrier (Cys247X-As) was very similar to the control, which agrees with the lack of disease observed in asymptomatic carriers.

### Whole transcriptome analysis from retinal organoids reveals altered expression of genes related to cell adhesion, POS, and cilia structures

**Fig. 9:**
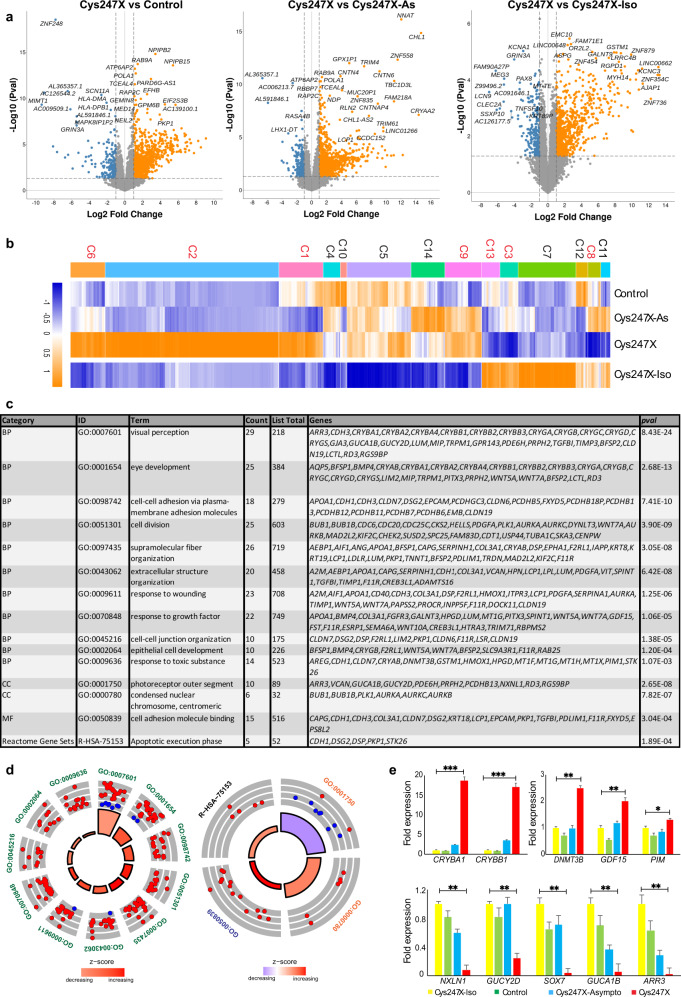
Identification of genes and pathways deregulated in D100 retinal organoids derived from *PRPF31* patient hiPSCs using whole transcriptome analysis. **a** Volcano plots of differentially expressed genes for Cys247X *vs* Control (left panel), Cys247X *vs* Cys247X-As (middle panel), and Cys247X *vs* Cys247X-Iso (right panel). Difference in gene expression (FC) is plotted on the x-axis (log2 scale), and *P* value (Pval) significance is plotted on the y-axis (log10 scale). Genes up- or downregulated are indicated in orange and blue, respectively. **b** Hierarchical clustering analysis of the DEGs between Cys247X-Iso and Cys247X with genes up- or downregulated by a factor ≥2 with *P* val ≤0.05 in D100 retinal organoids. The *z* score was derived from the average of the replicates for each experimental group. Blue: low expression; orange: high expression. The top row represents the 14 clusters, and selected clusters used for further pathway analysis are in red. **c** Table of the over-represented GO pathways of interest identified with Metascape using the DEGs from the selected clusters. The different categories of pathways are BP, Biological Process; CC, Cell Compartment; KEGG, Kyoto Encyclopedia of Genes and Genomes; MF, Molecular Function. **d** Circular visualization of selected GO enriched pathways. Downregulated genes (blue dots) and up-regulated genes (red dots) within each GO pathway of interest are plotted based on logFC. *z* score bars indicate if an entire biological process is more likely to be increased or decreased based on the genes it comprises. Green, Biological Process; orange, Cell Compartment; blue, Molecular Function. **e** Differential expression analysis by RT-qPCR of *CRYBA1, CRYBB1*, *DNMT3B*, *GDF15,* and *PIM* (upper panel) and of *NXLN1, GUCY2D, SOX7, GUCA1B,* and *ARR3* (lower panel) in Cys247X-Iso, Control, Cys247X-As, and Cys247X iRPE cells. Fold expression is relative to Cys247X-Iso. Values are mean ± SD, *n* = 3 samples of 10-12 retinal organoids from *N* = 3 independent differentiations. Data were normalized against the geometric average ΔCt of *18* *S* housekeeping gene. Statistical significance assessed using the Kruskal–Wallis test (*P < 0.05; **P < 0.01; ***P < 0.001).

To detect early transcriptional changes prior photoreceptor degeneration, we first performed whole transcriptome sequencing on D100 retinal organoids derived from Control, Cys247X-As, Cys247X-Iso, and Cys247X iPSCs. Filtering of the DEGs with an FC greater or lesser than 2, a *P* value lower than 0.05 and a minimum expression of five TPM in one group led to the identification of 567 DEGs for Cys247X *vs* Control, 704 DEGs for Cys247X *vs* Cys247X-As and 512 DEGs for Cys247X *vs* Cys247X-Iso (Fig. 9a). Hierarchical clustering based on *z* score allowed the selection of 7 clusters in which the 322 identified DEGs showed similar expression levels between Cys247X-Iso and Control as well as Cys247X-As but a different one in Cys247X (Fig. 9b and Supplementary Data 3). Pathway enrichment analysis of the selected DEGs revealed significant deregulation of genes related to visual perception, eye development and cell-cell adhesion, and extracellular organization (Fig. 9c, d and Supplementary Fig. 9). They also encompassed 14 genes coding for crystallin family members, known to be induced following retinal stress/injury^42^. Circular visualization of selected enriched pathways of interest indicated that a vast majority of them included up-regulated DEGs in Cys247X based on the calculated *z* score (Fig. 9d). The main pathway with a negative *z* score included genes coding for proteins located in the POS indicating that *PRPF31* mutations led to the downregulation of genes involved in the phototransduction. Based on our whole transcriptome analysis, the differential expression on few selected genes related to the most relevant identified pathways was confirmed by RT-qPCR. The expression level of these genes in organoids derived from the unaffected carrier (Cys247X-As) is relatively similar to the Control or intermediate between the Control and the affected carrier (Cys247X) (Fig. 9e).

As major changes started to be observed in D130 organoids, whole transcriptome sequencing was also performed on the different organoids at this stage using the same filtering parameters (FC2, *P* val ≤ 0.05, TMP ≥ 5), leading to the identification of 1567 DEGs for Cys247X *vs* Control, 2606 DEGs for Cys247X *vs* Cys247X-As and 632 DEGs for Cys247X *vs* Cys247X-Iso (Supplementary Fig. 10a, b). Hierarchical clustering based on *z* score allowed the selection of five clusters in which the 84 identified DEGs showed a similar expression level between Cys247X-Iso and Control as well as Cys247X-As but different in Cys247X (Supplementary Fig. 11 and Supplementary Data 3). Pathway enrichment analysis of the selected DEGs and circular visualization (Supplementary Fig. 10) revealed significant deregulation of genes related to pathways already identified at D100 (Fig. 9c). Altogether our results suggest that the early defects caused by *PRPF31* mutations concerned genes related to photoreceptor homeostasis and particularly outer segment formation/maintenance and composition of the extracellular matrix.

### Splicing defects in iRPE cells and retinal organoids carrying *PRPF31* mutations

**Fig. 10:**
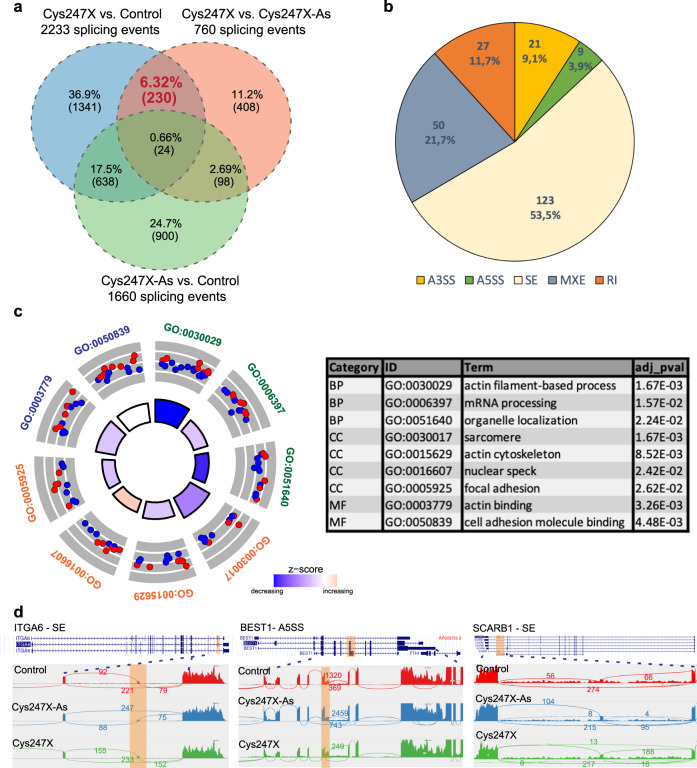
Identification of alternate splicing in hiPSC-derived RPE cells from *PRPF31* patients. **a** Venn diagrams comparing alternate splicing changes identified in iRPE cells and considered as significant (deltaPSI ≥ 10%, FDR ≤ 0.05) between Cys247X *vs* Control, Cys247X *vs* Cys247X-As and Cys247X-As *vs* Control. The number of alternate splicing events of interest are indicated in red and occurred in 152 genes. **b** Pie chart from rMATs analysis showing that a majority of splicing events corresponded to skipped exons. A5SS and A3SS, alternative 5′ and 3′ splice sites; SE, skipped exons; RI, retained introns; MXE, mutually exclusive exons. **c** Circular visualization of selected GO enriched pathways from the identified 152 genes with alternative splicing events. Down- (blue dots) and up-regulated genes (red dots) within each GO pathway are plotted based on logFC. *z* score bars indicate if an entire biological process is more likely to be increased or decreased based on the genes it comprises. Green, Biological Process; orange, Cell Compartment; blue, Molecular Function. The table shows pathway identity number (ID), associated pathway name (Term), gene count from the analyzed dataset and the adjusted *P* value (*adj_pval*). The different categories of pathways are BP, Biological Process; CC, Cell Compartment; MF, Molecular Function. **d** Sashimi plots for the indicated genes representing the alternate splicing events in iRPE cells derived from Control (green), Cys247X-As (blue) and Cys247X (red). Data are representative of the replicates for each experimental group. Orange highlights in the sashimi plots indicate the alternative splicing events and numbers correspond to the number of junction reads for each event. ITG6A: Integrin Subunit Alpha 6; BEST1: Bestrophin 1; SCARB1: Scavenger Receptor Class B Member 1.

Based on the role of *PRPF31* as a splicing factor, we used our whole transcriptome data from iRPE cells and retinal organoids at D100 to identify differences in splicing events between Cys247X patients (affected) and others (control, asymptomatic carrier and isogenic corrected). Using rMATS software and filtering parameters of a minimum PSI value of 10%, a deltaPSI of 10% and an FDR value lower than 0.05, we identified 2233 splicing events in iRPE cells for Cys247X *vs* Control, 760 for Cys247X *vs* Cys247X-As, and 1660 for Cys247X-As *vs* Control (Fig. 10a). We selected significant splicing events occurring in both comparisons for Cys247X *vs* Control, and Cys247X *vs* Cys247X-As but not in Cys247X-As *vs* Control. Such approach led to the identification of 230 splicing events altering a total of 152 unique genes (Supplementary Data 4). More than half of them corresponded to exon skipping events and around 22% to mutually exclusive exons (Fig. 10b). GO enrichment analysis of the biological process, cell compartment, and molecular function showed that Cys247X iRPE cells had significant differential exon usage for transcripts involved in the cytoskeleton (actin filament-based process, sarcomere, actin cytoskeleton, cell adhesion, and actin binding) (Fig. 10c). These observations corroborate with the defective RPE phenotype observed in Cys247X iRPE cells. Interestingly, alternative splicing occurred also on transcripts related to mRNA processing including four ribosomal binding motif protein-family members, suggesting a role of the PRPF31 splicing factor in the regulation of the mRNA processing machinery. Using Sashimi plots (Fig. 10d), we confirmed splicing defects for *ITGα6* (skipped exon) and *BEST1* (alternative 5′ splice sites), important genes associated with RPE homeostasis^43^, and for *SCARB1* (skipped exon), recently described as involved in internalization of OS for RPE-mediated phagocytosis^44^.

**Fig. 11:**
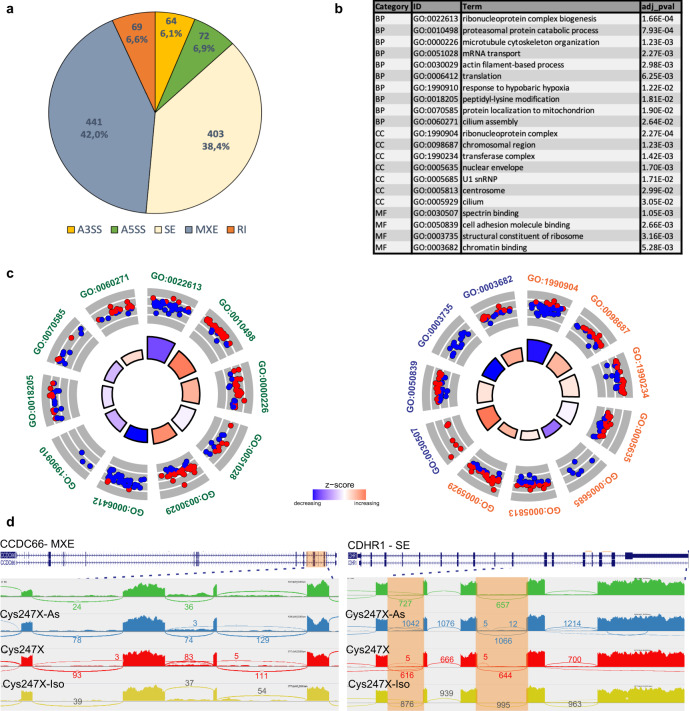
Identification of alternate splicing in D100 retinal organoids derived from *PRPF31* patient hiPSCs. **a** Pie chart from rMATs analysis showing that a majority of splicing events in retinal organoids corresponded to skipped exons and mutually exclusive exons. A5SS and A3SS, alternative 5′ and 3′ splice sites; SE, skipped exons; RI, retained introns; MXE, mutually exclusive exons. **b** Table showing pathway identity number (ID), associated pathway name (Term), gene count from pathways analysis and the adjusted *P* value (adj_pval). The different categories of pathways are BP, Biological Process; CC, Cell Compartment; MF, Molecular Function. **c** Circular visualization of selected GO enriched pathways from the identified 600 genes with alternative splicing events (total of 1049 splicing events) between Cys247X-Iso and Cys247X retinal organoids at D100. Down- (blue dots) and up-regulated genes (red dots) within each GO pathway are plotted based on logFC. *z* score bars indicate if an entire biological process is more likely to be increased or decreased based on the genes it comprises. Green, Biological Process; orange, Cell Compartment; blue, Molecular Function. **d** Sashimi plots for the indicated genes representing the alternate splicing events in retinal organoids at D100 derived from Control (green), Cys247X-As (blue), Cys247X (red) and Cys247X-Iso (yellow). Data are representative of the replicates for each experimental group. Orange highlights in the sashimi plots indicate the alternative splicing events and numbers correspond to the number of junction reads for each event. CCDC66: Coiled-Coil Domain Containing 66; CDHR1: Cadherin Related Family Member 1.

For retinal organoids, we used the same filtering parameters on rMATS data to compare cells derived from affected patient (Cys247X) and isogenic corrected (Cys247X-Iso) iPSCs. We also filtered out genes with a TPM value lower than 5 in one group to keep splicing events on genes expressed at a significant level in the target cells. As such, we identified 1049 differential splicing events corresponding to 600 unique genes (Fig. 11a and Supplementary Data 5). Among the five types of events detected, a large majority corresponded either to exon skipping (38%) or to mutually exclusive exons (42%). GO enrichment analysis of biological process, showed that Cys247X retinal organoids had significant differential exon usage for transcripts involved in mRNA regulation (ribonucleoprotein complex biogenesis, mRNA transport, translation), cytoskeleton (microtubule cytoskeleton organization, actin filament) and cilia (cilium assembly) (Fig. 11b, c). Identified GO enrichment analysis for cell compartment and molecular function confirmed the splicing alteration for transcripts related to previously identified biological processes. Using Sashimi plots (Fig. 11d), we confirmed splicing defects for the integrin *CCDC66* (MXE) and *CDHR1* (skipped exon) genes which mutations have been associated with retinopathies^45,46^. Using the same filtering parameter applied for D100, we identified 513 differential splicing events corresponding to 307 unique genes comparing D130 organoids derived from Cys247X and Cys247X-Iso iPSCs (Supplementary Fig. 10c, d and Supplementary Data 5). A large majority of events still corresponded to exon skipping (39%) but the number of retention introns events was notably increased (from 7% at D100 to 27% at D130) at the expense of mixed exons events (from 42% to 13.5%). Nevertheless, and as for D100 organoids, GO enrichment analysis revealed a significant differential exon usage in D130 Cys247X organoids for transcripts involved in cytoskeleton, cilia and numerous pathways related to mRNA and RNA splicing (Supplementary Fig. 10). Overall, exon usage analysis in iRPE cells and retinal organoids led to the identification of altered transcripts belonging to common pathways related to cytoskeleton and mRNA regulation. In retinal organoids, we specifically identified splicing defects occurring in transcripts coding for ciliary protein.

## Discussion

Due to the ubiquitous nature of RNA splicing, there is much debate regarding the underlying disease mechanism by which mutations in *PRPF31* lead to a retina-specific phenotype. Several studies using human post-mortem retinas or patient lymphoblast cell lines showed the absence of specific PRPF31 isoforms in the retina, as well as both steady-state levels of snRNAs and processed pre-mRNAs highest in the retina, indicating the requirement of a particularly elevated splicing activity in this tissue^22^. Furthermore, the identification of which retinal cell type (RPE and/or photoreceptors) is primarily affected by mutations in the RNA splicing factors lead to a scientific debate^13,47^. To gain insights into *PRPF31*-related RP, we characterized the cellular phenotype of RPE cells and retinal organoids derived from both clinically asymptomatic carriers and affected *PRPF31* patients carrying two different mutations. We demonstrated that *PRPF31* mutations affect both RPE cells and photoreceptors, suggesting that photoreceptor degeneration could be due to a combination of photoreceptor-specific autonomous mechanisms and a consequence of RPE alteration.

The different *PRPF31*-mutated iPSCs generated in our study expressed pluripotent markers and presented classical properties of human pluripotent stem cells, indicating that mutations in the ubiquitous *PRPF31* gene did not affect the reprogramming of the pluripotent state per se. Furthermore, we were able to generate RPE cells and pseudo-laminated retinal organoids from several iPSC lines carrying different *PRPF31* mutations, confirming that the differentiation process is not impaired, as recently reported for different *PRPF8* and *PRPF31* iPSCs^29,30,48^.

However, the RPE cells differentiated from *PRPF31*-mutated iPSC lines revealed several important morphological changes including disorganized cell-to-cell contact, absence of desmosomes and basal membranes, and impaired polarization that could explain their altered TER and reduced phagocytic activity. These observations are consistent with adhesion and polarity defects and a significant decrease in phagocytosis reported in primary RPE cultures of *Prpf31*^*+/-*^ mice and in a human RPE cell line in which *PRPF31* expression was repressed by RNA silencing^24^, as well as in RPE cells derived from iPSCs carrying different *PRPF31* mutations^29,30^. In contrast to our data, Buskin et al. reported a disruption of phagocytosis only in RPE cells derived from iPSCs of *PRPF31* patient with important clinical severity^29^. The incomplete penetrance of *PRPF31*-related RP could explain these correlations between cellular phenotype and genotype, but further investigations by including more patients are required to validate this hypothesis.

All the *PRPF31*-mutated iPSC lines used in this study successfully generated 3D laminated retinal organoids with rod and cone photoreceptors. Strikingly, we observed a significant decrease in the number of photoreceptors, first of rods after 130 days, followed by a decrease in the number of cones after 175 days, with a disorganization and thinning of the presumptive ONL in all organoids derived from affected *PRPF31* patients iPSCs. The presence of relatively early NRL-positive rods, cone arrestin-positive cones, and other late-onset cellular markers such as PKCα-positive bipolar cells and Müller glial cells up to 200 days of differentiation, confirms that it is not linked to a developmental delay, but actually reflects a death of differentiated photoreceptor cells corresponding to the human RP phenotype. Compared to the situation observed in *PRPF31* patients, photoreceptor cell death is observed at an earlier ‘age’, as has been reported in retinal organoids derived from iPSC lines carrying a mutation in other RP genes such as *RPGR*^49^ or *RP2*^50^. Sources of stress inherent in the in vitro organoid technology, due to the absence of an opposing RPE monolayer or to the difficulty of supplying nutrients and oxygen due to the lack of vascularization of the organoids, could explain the accelerated onset of the disease phenotype. Nevertheless, our iPSC-derived disease model clearly shows a degeneration of rods followed by a secondary loss of cones, recapitulating the disease progression in splicing factor-related RP. A severe decrease in photoreceptor cell layer has been reported in the zebrafish by using *Prfp31* morphants^51^, and the presence of apoptotic nuclei was previously observed in photoreceptors within late retinal organoids derived from *PRPF31* patients iPSCs^29^. However, no defective photoreceptor phenotype was reported in different mouse models of splicing factors-linked RP^23,24,47^. It is not known why *Prpf31* mutant mice have only an RPE degenerative phenotype and do not present the key phenotype of *PRPF31*-related RP patients, *i.e*. the photoreceptor cell death. The higher level of Prpf31 expression in murine RPE cells than in the neural retina could partly explain this observation^52^.

We have generated iPSCs from two patients carrying different *PRPF31* mutations and observed a similar cell phenotype in iRPE cells and retinal organoids in both cases. These *PRPF31* mutations lead to the occurrence of a premature stop codon that probably results in null alleles and decreases amounts of functional PRPF31 protein in affected cells, as confirmed by western blot in RPE cells and retinal organoids. Notably, we generated isogenic CRISPR/Cas9-repaired iPSCs to confirm specificity of the genotype-phenotype relationships. Precise gene repair of *PRPF31* locus (exon 8) generated isogenic clones in which all the cellular and structural deficiencies observed in affected patient-derived RPE cells and retinal organoids were reversed, demonstrating that the phenotypic differences are directly linked and specific to the presence of the *PRPF31* mutation. Consistently, PRPF31 protein expression levels in RPE cells and retinal organoids after correction were identical to that of the control, confirming that PRPF31 below a certain threshold triggers disease onset.

Haploinsufficiency due to the loss of function of the mutated protein or degradation of mutant mRNA nonsense-mediated decay has been proposed as the major cause of RP linked to *PRPF31* mutations^16,17,53^. Investigation of PRPF31 protein abundance in lymphoblastoid cell lines derived from patients carrying different mutations showed a decrease in their expression levels^17,53^. Our results showed that the amount of PRPF31 protein is significantly reduced in the retinal cell types of patients compared to those derived from control patients, as observed in retinal cells derived from iPSCs carrying other *PRPF31* mutations^29,30^. The defective phenotypes observed in RPE cells and retinal organoids is thus correlated to this reduced PRPF31 expression level. This correlation was validated in our study by phenotype analysis of retinal derivatives from asymptomatic patient iPSCs, demonstrating the absence of a defective phenotype in both RPE cells and retinal organoids.

Since most of the currently identified mutations in *PRPF31* cause disease via haploinsufficiency, one of the most obvious potential therapeutic strategy is gene augmentation, which has the advantage of not being mutation-specific and can therefore address the large number of mutations described for *PRPF31*^13^. Our CRISPR/Cas9 KI approach in patient iPSCs demonstrated that insertion of a functional copy of *PRPF31* into a specific safe locus of iPSCs from *PRPF31* RP patients restored the expression levels of the PRPF31 protein and totally prevented the defective phenotype of RPE cells and photoreceptors in retinal organoids. Although CRISPR/Cas9-mediated in situ gene editing is becoming a popular methodology, we also achieved a more conventional approach for future clinical trials using AAV vectors. The use of the AAV2-7m8 variant optimized for photoreceptor transduction^38,54^ restored PRPF31 protein expression and thus prevented rod and cone photoreceptor degeneration in mature retinal organoids transduced before the first signs of degeneration. This result opens up new perspectives for the treatment of *PRPF31*-related RP. It would be interesting to determine whether this strategy is also effective at later stages of the disease, when photoreceptor degeneration has already been initiated, in order to identify a time window for future gene therapy intervention. As the same rescue effect was recently reported in RPE cells derived from iPSCs, where one copy of *PRPF31* was deleted by CRISPR/Cas9^30^, the use of an AAV vector targeting both photoreceptors and RPE cells may be advantageous to simultaneously target both affected cell types. Establishing a safe dose range for PRPF31 overexpression is an important future milestone prior moving to the clinic, since it is conceivable that higher ratios of PRPF31 production could adversely affect the splicing machinery and potentially photoreceptor homeostasis and survival. Furthermore, to minimize the risks associated with retinal detachments, it seems essential in the future to use a vector that can successfully deliver the gene to the outer retina via intravitreal injection, as it is possible with the 7m8 variant^54^ presented here.

Ultimately, knowledge of the molecular pathways affected by mutations in *PRPF31* in iPSC-derived retinal cells should help developing therapies to slow or reverse RP. In this context, the in-depth transcriptomic study of iRPE cells and retinal organoids allowed us to identify differentially expressed and/or spliced transcripts correlated with the phenotypic alterations observed at the cellular level. Mainly genes are involved in cell morphogenesis, cell adhesion, extracellular matrix composition, and apoptosis for both RPE cells and organoids, as well as more “photoreceptor-specific pathways” for retinal organoids, such as visual perception, POSs, and cilia. Notably, impaired splicing events are observed in different genes involved in RNA processing pathways including the splicing program itself both in iRPE cells and retinal organoids, suggesting that exacerbation of splicing deficiencies contributes to the defective phenotype observed in retinal cells and thus probably in *PRPF31*-related RP patients. These results are in agreement with those obtained previously in RPE cells and retinal organoids derived from iPSCs of patients carrying different *PRPF31* mutations^29^, or on human retinal explants where *PRPF31* has been silenced^31^. Identification of the mis-spliced transcripts responsible for the defective phenotype associated with *PRPF31* mutations in iPSC retinal derivatives could also help to develop pharmacological therapies to slow or reverse RP.

In summary, our study provides insights into the use of iPSC-derived cells and organoids for retinal disease modeling, with a phenotype related to mutations in splicing factors and in an effort to identify potential pathomechanisms of retinal degeneration. Furthermore, we highlighted how the restoration of PRPF31 expression can reverse this phenotype, paving the way for a potential mutation-independent AAV-based gene therapy for the treatment of *PRPF31*-related RP.

## Methods

### Human subjects

All skin biopsies used in the study for fibroblasts isolation were obtained from patients who provided written informed consent to take part in the study under the approval of French regulatory agencies: CPP Ile de France (2012-A01333–40; P12-02) and the French Agency for the Safety of Health Products (ANSM) (B121362-32).

### Human fibroblast cultures and reprogramming

Human fibroblasts were cultured and reprogrammed using the CytoTune Sendai reprogramming vectors Oct4, Klf4, Sox2, and c-Myc (Thermo Fisher Scientific) as previously reported^37^. The emergent iPSC colonies were picked under a stereomicroscope and expanded onto mitomycined human foreskin feeder layers. After the generation of a frozen stocks, iPSCs were preferentially adapted and cultured in feeder-free conditions^34^. After ten passages, the clearance of the exogenous reprogramming factors and Sendai virus genome was confirmed by qPCR following the manufacturer’s instructions (Thermo Fisher Scientific). The absence of mycoplasma contamination was verified by the MycoAlert™ Mycoplasma Detection Kit (selective biochemical test of mycoplasma enzymes) according to the manufacturer’s instructions (Lonza).

### iPSC culture

Human iPSCs were routinely cultured on truncated recombinant human vitronectin (rhVTN-N)-coated dishes with mTeSR^TM^1 medium (StemCell^TM^ Technologies) at 37 °C in a standard 5% CO_2_/95% air incubator with a daily medium change. iPSCs were passaged once a week with the enzyme-free Gentle cell dissociation reagent (StemCell^TM^ Technologies) at RT for 6 min. Detached cell aggregates were collected in mTeSR^TM^1 medium and carefully pipetted up and down to obtain uniform suspensions of cell aggregates that are transferred to fresh rhVTN-coated plates in a 1:10 to 1:60 ratio. iPSCs were cryopreserved with the CryoStor CS10 cell freezing medium (StemCell^TM^ Technologies).

### Karyotype analysis

Actively growing hiPSC colonies (80% confluency) were treated with colchicin (20 mg/ml; Eurobio) for 90 min at 37 °C. Cells were dissociated with 0.05% trypsin-EDTA and incubated in 75 mM KCl (Sigma-Aldrich) for 10 min at 37 °C, before fixation with 3:1 methyl alcohol/glacial acetic acid. Fixed cells were hybridized overnight at 37 °C with a denatured “cocktail painting mFISH” probe (MetaSystems). Slides were washed in successive baths of 1× SSC and 0.4× SSC, and nuclei were stained with 250 ng/ml 4’,6-diamidino-2-phenylindole (DAPI). Biotinylated probes were revealed using a Cy5 MetaSystems B-tect detection kit (MetaSystems). 10 to 20 metaphases were captured using a Zeiss Z1 fluorescence microscope equipped with a UV HBO 100-W lamp coupled to an AxioCam camera (Carl Zeiss). All of the analyzed metaphases were karyotyped using the MetaSystems Isis software (MetaSystems).

### In vitro and in vivo three germ layer differentiation

To validate the pluripotency of hiPSC lines, a teratoma-formation assay was performed as previously described^55^ with slight modifications. Briefly, 1 × 10^6^ to 2 × 10^6^ cells were injected into the rear leg muscle of 6-week-old NOD Scid gamma (NSG) mice (Charles River). Animals were killed according to the recommendations of our local ethical and animal care committee (Institut de la Vision - Authorization B-75-12-02 delivered by the Ministry of Agriculture). After 9–10 weeks, teratomas were dissected and fixed in 4% paraformaldehyde. Samples were then embedded in paraffin, and sections were stained with hematoxylin/eosin.

Pluripotent state of iPSCs was also assessed in vitro using the STEMdiff^TM^ trilineage differentiation kit (StemCell^TM^ Technologies) according to the manufacturer’s instructions. Briefly, iPSCs were dissociated into single cells using a combination of Gentle Cell Dissociation Reagent (StemCell^TM^ Technologies) for 7 min at 37 °C and Accutase (Gibco) for 4 min at RT. Cells were plated at a density of 50 cells/cm^2^ (mesoderm lineage) or 200 cells/cm^2^ (endoderm and ectoderm lineages) on rhVTN-N-coated 12-well plates containing mTeSR^TM^1 medium supplemented with RevitaCell^TM^ Supplement. After 24 h, media were aspirated and cells were cultured with appropriate STEMdiff^TM^ trilineage medium for 5 (mesoderm and endoderm lineages) or 7 days (ectoderm lineage) with a daily medium change, before cell fixation and assessment of differentiation for each germ layer by immunofluorescence using specific markers (Supplementary Data 6).

### Mutation screening

Genomic DNA from control and patients iPSCs were extracted using the Nucleospin Tissue Kit (Macherey-Nagel) following the manufacturer’s instructions. PCR amplification flanking *PRPF31* mutations in exons 4 or 8 (Supplementary Data 6) was performed using HOT FIRE Pol DNA Polymerase (Solis BioDyne). PCR products were sequenced using BigDye® Terminator v1.1 Cycle Sequencing Kit (Thermo Fisher Scientific) on a 3730 DNA analyzer (Applied Biosystems).

### CRISPR/Cas9 correction of the Cys247X *PRPF31* mutation

Correction of the *PRPF31* mutation in the Cys247X iPSCs was achieved by using the CRISPR/Cas9 system in combination with ssODN as a homologous template covering the mutation site. The CRISPOR website tool (http://crispor.tefor.net) was used to design the sgRNA sequences and predict off-targets. The sgRNA (Supplementary Data 6), which targets only the mutated sequence and is predicted to have low off-targets, was chosen and synthetized using Alt-R guide RNA modifications (Integrated DNA Technologies). The ssODN template with wild-type *PRPF31* sequence was designed manually with 91-bp homology arms on each side of the mutation region (Supplementary Data 6). The Cas9 ribonucleoprotein (RNP) was prepared by incubating Alt-R Cas9 enzyme (Integrated DNA Technologies) with the sgRNA at RT for 15 min following the manufacturer’s instructions and the RNP complex was co-transfected in iPSCs carrying the Cys247X mutation using the Lipofectamine Stem Reagent (Thermo Fisher Scientific) following the manufacturer’s instructions. ssODN was added 24 h later using the Lipofectamine RNAiMAX Transfection Reagent (Thermo Fisher Scientific) according to the manufacturer’s instructions. Three days after the transfection, iPSCs were dissociated in single cells using a combination of Gentle Cell Dissociation Reagent for 7 min at 37 °C and Accutase for 4 min at RT. Cells were plated at densities of 200, 400, or 600 cells/cm^2^ on a rhVTN-N-coated 10-cm diameter culture dish containing 10 ml of mTeSR^TM^1 medium supplemented with RevitaCell^TM^ Supplement. After one week, colonies were manually picked up and transferred to a cell culture 96-well cell culture plate. Sub-confluent iPSCs were split into two 96-well plates to allow further culture expansion and DNA isolation using QuickExtraxt^TM^ DNA Extraction Solution (Lucigen). The PCR was composed of 32 cycles including three successive steps at 95 °C, 60 °C, and 72 °C, respectively, for 30 s, 30 s, and 2 min. The genotype was finally visualized after migration of the DNA in a 3% agarose gel (Supplementary Data 6). After validation by sequencing, corrected IPSC lines were selected, pluripotent status was validated by immunostaining for pluripotency markers, and the genomic stability was assessed by detection of recurrent genetic abnormalities using the iCS-digital^TM^ PSC test by Stem Genomics (Montpellier, France).

Off-target sequences were predicted using the CRISPOR tool website (http://crispor.tefor.net). The sgRNA off-target sequence was blasted against the human genome reference (https://blast.ncbi.nlm.nih.gov/Blast.cgi). Capture intervals of predicted off-target sites were expanded by ~500 bp in both the 5′ and 3′ directions to design specific primers ensuring accurate detection of any genetic lesion occurring at or near the selected sites. The PCR products were then sequenced to check the off-target effects of sgRNA (Supplementary Data 6).

### Generation of the AAVS1::CAG-P PRPF31 iPSC line

To construct a donor plasmid for the expression of *PRPF31* under the control of the CMV early enhancer/chicken β actin (CAG) promoter from the AAVS1 locus, AAVS1-cAGGS-EGFP (Addgene #22212)^56^ containing the puromycin-resistant gene and EGFP under the control of a CAG promoter between AAVS1 homology arms of 800 bp each (HA-L and HA-R) was modified. The sequence of human *PRPF31* cDNA (NM_015629) was subcloned by a XbaI/MluI digestion between AAVS1 homology arms of 800 bp each (HA-L and HA-R). We used pX330-U6-Chimeric_BB-CBh-hSpCas9 (addgene#42230)^57^ to construct the CRISPR/Cas9 vector by annealing and ligating in BbsI sites an oligonucleotide pair (5′-CACCGGGGCCACTAGGGACAGGAT-3′ /3′-CCCCGGTGATCCCTGTCCTACAAA-5′) encoding a 20-nt AAVS1 guide sequence according to Ran et al.^58^. Plasmids are prepared using the Plasmid MidiPrep kit (Macherey-Nagel), verified by restriction digestion and gel electrophoresis for identity and integrity, and concentrations determined by NanoDrop detection (Thermo Fisher Scientific) before storage at 1–2 µg/µl in sterile Tris buffer (pH 8.0). The generation of stable cell clones was performed as previously described^59^. hiPSC lines carrying *PRPF31* mutations were grown for 24 h in six-well plates before overnight transfection at 37°C with CRISPR/Cas9 and donor plasmids using FuGene HD (ratio 1:1:5; Promega). Two days after transfection, selection of recombinant cells was started in the presence of 0.25 μg/ml puromycin (Thermo Fisher Scientific) for the first 48 h and the concentration of puromycin was then increased to 0.5 μg/ml until picking of single colonies. Genomic DNA from puromycin-resistant clones was extracted using the NucleoSpin Tissue kit (Macherey-Nagel) according to the manufacturer’s instructions. Correct reporter integration was evaluated by PCR using a forward primer upstream to the AAVS1 left arm of recombination, and a reverse primer either within the reporter cassette or in the AAVS1 right homologous arm. The PCR was composed of 32 cycles including three successive steps at 95 °C, 60 °C, and 72 °C, respectively, for 30 s, 30 s, and 2 min. The genotype was finally visualized after migration of the DNA in a 1.2% agarose gel. After validation by sequencing heterozygous integrated iPSC lines were selected, the pluripotent status was validated by immunostaining for pluripotency markers, and the genomic stability was assessed by detection of recurrent genetic abnormalities using the iCS-digital^TM^ PSC test by Stem Genomics.

### Retinal differentiation of hiPSCs

Retinal cell differentiation was performed according to a previously described protocol^34,35^. Briefly, hiPSCs were expanded to 70–80% confluence in 6-cm diameter culture dishes coated with rhVTN-N in mTeSR^TM^1 medium. At this time, defined as day 0 (D0), hiPSC colonies were cultured in the chemically defined TeSR™-E6™ medium (StemCell^TM^ Technologies). After 2 days, the medium was switched to E6N2 medium composed of TeSR™-E6 medium, 1% N2-supplement-A (StemCell^TM^ Technologies), 10 units/ml penicillin, and 10 µg/ml streptomycin (Thermo Fisher Scientific). The medium was changed every 2–3 days.

On D28, identified self-formed retinal organoids were isolated using a needle with the surrounding cells, and cultured in ultra-low attachment 24-well plates (Corning) as floating structures in the ProB27 medium supplemented with 10 ng/ml of animal-free recombinant human FGF2 (Peprotech) and half of the medium was changed every 2–3 days. The ProB27 medium is composed of chemically defined DMEM:Nutrient Mixture F-12 (DMEM/F-12, 1:1, l-Glutamine), 1% MEM non-essential amino acids (Thermo Fisher Scientific), 2% B27 supplement (Thermo Fisher Scientific), 10 units/ml penicillin and 10 µg/ml streptomycin. At D35, retinal organoids were cultured in the absence of FGF2 in the ProB27 medium with 10% FBS (Thermo Fisher Scientific) and 2 mM of Glutamax (Thermo Fisher Scientific) for the next several weeks. To evaluate the maturation of photoreceptors, at D84, the retinal organoids were cultured in the ProB27 medium with 2% B27 supplement without vitamin A (Thermo Fisher Scientific) until D200.

For RPE culture, at D28 after retinal organoid isolation, the medium of 6-cm diameter dishes were switched to DMEM:Nutrient Mixture F-12 (DMEM/F-12, 1:1, L-Glutamine), with 1% N2-supplement-A, 1% MEM non-essential amino acids, and 10 units/ml penicillin and 10 µg/ml streptomycin (RPE medium). The medium was changed every 2–3 days until the apparition of regions of pigmented cells. Identified patches of pigmented cells were then picked up with a needle, replated onto new cell dishes previously coated with Geltrex® (Thermo Fisher Scientific), and expanded in the RPE medium. At confluency, iPSC-derived RPE cells can be dissociated using TrypLE Express (Thermo Fisher Scientific) and plated in new coated dishes for amplification or banking.

### Cell or tissue fixation and cryosection

RPE cells were fixed for 10 min with 4% paraformaldehyde (PFA) at 4 °C and then washed twice in phosphate-buffered saline (PBS) before immunostaining. Retinal organoids were fixed for 15 min in 4% PFA at 4 °C and then washed twice in PBS. Structures were incubated at 4 °C in PBS/30% sucrose (Sigma-Aldrich) until they all sank to the bottom of the well. Structures were embedded in PBS, 7.5% porcine skin gelatine (Sigma-Aldrich), 10% sucrose solution, frozen in isopentane at −70 °C and stored at −80 °C. 10 µm-thick cryosections were collected (Microm Cryostat) and stored at −80 °C.

### Immunostaining, imaging, and cell counting

Immunofluorescence was performed either on fixed cells or on retinal organoid cryosections. Slides were then incubated for 1 h with a blocking solution (PBS, 0.2% gelatine, and 0.1% Triton X-100) at RT. Incubation with the primary antibody (Supplementary Data 6), appropriately diluted in blocking solution, was performed alternatively for 1 h at RT or overnight at 4 °C. Slides were washed four times in PBT (PBS with 0.1% Tween-20) and then incubated for 1 h at RT with appropriate secondary antibody conjugated with either AlexaFluor-488, 594 or 647 (Interchim) diluted at 1:600 in the blocking solution. To counterstain the nuclei, slides were washed twice in PBT, and incubated with DAPI diluted at 1:1000 in PBS. Finally, slides were washed two times in PBS before mounting with Fluoromount-G (Southern Biotech).

Fluorescent staining signals were captured with a DM6000 microscope (Leica microsystems) equipped with a CDD CoolSNAP-HQ camera (Roper Scientific) or using an Olympus FV1000 confocal microscope equipped with standard PMTs, high-sensitivity GaAsP detectors. AlexaFluor-488, AlexaFluor-594, and AlexaFluor-647 were excited by using a 488 nm argon ion laser line, 559 nm, and 635 nm laser diodes lines, respectively. Control of the confocal microscope and image acquisitions were conducted by using the Olympus Fluoview software version 4.2 at a resolution of 1024 × 1024 pixels, with a scan rate of 8–10 μs pixel^−1^. Images were acquired sequentially, line by line, in order to reduce excitation and emission crosstalk, step size was defined according to the Nyquist-Shannon sampling theorem. Exposure settings that minimized oversaturated pixels in the final images were used. Twelve-bit images were processed with ImageJ or FIJI, Z-sections were projected on a single plane using maximum intensity under the Z-project function.

Quantification of the number of total cells in the presumptive ONL (DAPI) and cells immunoreactive for specific markers was performed by manual counting using the FIJI/ImageJ software. A minimum of nine retinal organoids from three independent differentiation experiments were counted for each condition. The counted areas corresponded to one-quarter of the area of the retinal organoid cryosection.

To analyze RPE cell shape parameters, images of cells outlined by ZO-1 were first modified using several filtering steps (Maximum filter, Gaussian Blur) and binarized in order to apply a Voronoi function. Then, a threshold was performed, converted as a mask, inverted, and connected-components labeling was done before applying the Glasbey LUT to colorize each Voronoi cell. The MorpholibJ plugin^60^ was used to perform a second round of filtering (Flood Fill Components Labeling, Dilate Labels and Exclude Labels on Edges), and analyze the selected cell shape parameters of interest (Area, Convexity, Geodesic diameter). Area equals the actual number of pixels, Convexity, the ratio Convex Perimeter/Perimeter, and Geodesic Diameter corresponds to the largest geodesic distance between two points within an individual ZO-1 outlined RPE cells. Distribution data were extracted with BAR plugin^61^ and plotted with Prism 7 (GraphPad software).

### Scanning electron microscopy (SEM) and transmission electron microscopy (TEM)

RPE cells cultured on Transwell® (Corning) were fixed with 2% glutaraldehyde (and 1% PFA for TEM-analyzed samples) in 0.1 M PBS (pH 7.4), for 1 h at RT and washed in PBS. For SEM, samples were dehydrated in a graded ethanol series and dried by rinsing with hexamethyldisilane. Samples were mounted on an aluminum stub with a carbon scotch disc. They were then sputter coated with gold and were observed by the Cambridge Instruments Stereoscan 260 SEM (Leica microsystems). For TEM, samples were post-fixed in 1% osmium-bidistilled water for 1 h at RT. After bidistilled water washes, samples were dehydrated in increasing concentrations of ethanol. Samples were then infiltrated in 1:1 ethanol:epon resin for 1 h and then 100% epon resin for 48 h at 60 °C for polymerization. 70 nm-thick sections were cut with an ultracut UCT microtome (Leica microsystems) and picked up on copper rhodium-coated grids. Grids were stained for 2 min in Uranyless (Delta Microscopies) and for 5 min in 0.2% lead citrate, and further analyzed on an EM 912 OMEGA electron microscope (Carl Zeiss) at 80 kV and images were captured with a digital camera (Side-Mounted TEM CCD, Veleta 2kx2k).

### Western blot analysis

1 × 10^6^ hiPSC-derived RPE cells or 10 retinal organoids were lysed in 100 μl of RIPA Lysis Buffer (1% Triton 100×, 1% Sodium deoxycholate, 0.1% sodium dodecyl sulfate (SDS), 50 mM HEPES (pH 7,4), 150 mM NaCl, 10% Glycerol, 1.5 mM MgCl_2_, 1 mM EGTA) containing protease and phosphatase inhibitors (Sigma-Aldrich). The mix was then vortexed for 30 min in a cold room. Homogenates were centrifuged (4 °C, 10 min, 12,000×*g*). Supernatants were then collected to determine protein concentrations using a Bradford protein assay with the Infinite M1000 PRO (Tecan). Afterwards, 12 μg of proteins were denatured for 5 min at 98 °C before migration on an SDS-polyacrylamide gel (Mini-PROTEAN TGX Precast Gel, Bio-Rad) for 55 min at 150 V and transferred to a nitrocellulose membrane (Trans-blot Turbo Transfer 0.2 μm nitrocellulose, Bio-Rad). After transfer, membranes were saturated using a blocking solution (PBS, 0.1% Tween-20 with 5% non-fat powder milk) for 1 h at RT, and incubated with the primary antibody (Supplementary Data 6) overnight at 4 °C. After 4 wash with PBS-Tween 0.1%, membranes were incubated 1 h at RT with the respective anti-HRP mouse or rabbit secondary antibodies (Jackson, 1:10000). Revelation was performed using the Pierce ECL Plus (Thermo Fisher Scientific) according to the manufacturer’s recommendations. Chemiluminescence was detected using the Fusion Fx7 (Vilber Lourmat) and signals were quantified with the ImageJ software. A minimum of 3 independent differentiation experiments were used for each condition and all blots from the same experiment were processed in parallel. Uncropped blots are shown in Supplementary Data 7.

### Measurement of the transepithelial resistance

The transepithelial resistance (TER) of hiPSC-derived RPE cells seeded at 10^5^ cells on the porous membrane (Transwell® inserts, Corning) coated with Geltrex was performed using an EVOM Epithelial Voltohmeter (World Precision Instruments) by inserting the shorter and longer tips of the STX2 electrode (World Precision Instruments) in the apical and basolateral chambers, respectively. The TER was measured one time per week and values were then reported in Ω cm^2^, resistance reading of the blank (Transwell® insert only filled with RPE medium) being subtracted from each RPE cell resistance reading measurements.

### Phagocytosis assay

POS were purified from porcine eyes and covalently labeled with fluorescent dye by incubation with 0.1 mg/ml FITC (Isomer-1) according to established procedures^62^. Each well was layered with 100 μl of DMEM/F-12 (Thermo Fisher Scientific) containing 1 × 10^6^ POS particles and was incubated at 37 °C for 3 h before three washes with PBS containing 1 mM MgCl_2_ and 0.2 mM CaCl_2_ (PBS-CM). For exclusive detection of internalized particles, fluorescence of surface-bound FITC-POS was selectively quenched by incubation in 0.2% trypan blue in PBS-CM during 10 min. Cells were then fixed by incubation in ice-cold methanol for 10 min, rehydrated 10 min in PBS-CM, and incubated with DAPI for 10 min at RT. Fluorescent signals were quantified with the Infinite M1000 Pro (Tecan) microplate reader. The specific fluorescence signal of bound particles is obtained by subtracting the fluorescence signal of internalized particles (trypan blue quenching) from the total fluorescence signal. The human ARPE-19 cell line was used as a positive control for phagocytic activity and hiPSC-derived RPE cells in the absence of POS were used as a negative control.

### RNA extraction and Taqman assay

Total RNAs were extracted using the NucleoSpin RNA XS kit (Machery-Nagel), according to the manufacturer’s protocol. RNA yields and quality were determined using a NanoDrop ND-1000 Spectrophotometer (Thermo Fisher Scientific) before storage at −80 °C. cDNAs were synthesized from 250 ng of mRNA using the QuantiTect reverse transcription kit (Qiagen) following manufacturer’s recommendations. Synthesized cDNAs were diluted 1:20 in DNAse-free water before performing quantitative PCR. qRT-PCR analyses were performed on Applied Biosystems real-time PCR systems (7500 Fast System) using a custom TaqMan® Gene expression Master Mix (Thermo Fisher Scientific). All primers and MGB probes labeled with FAM for amplification were purchased from Thermo Fisher Scientific (Supplementary Data 6). Plates were run according to the following thermal cycling conditions: 2 min at 50 °C, 10 min at 95 °C; 40 repeats of melting 15 sec at 95 °C, and annealing/extension 1 min at 60 °C. All samples were normalized against the *18* *S* housekeeping gene. Quantification of gene expression was based on the ΔCt Method. Average values were obtained from at least three independent biological experiments and two technical replicates.

### Whole transcriptome sequencing (RNA-Seq) and data analysis

Total RNAs were extracted using the NucleoSpin RNA XS kit according to the manufacturer’s protocol. RNA yields and quality were determined using a NanoDrop ND-1000 Spectrophotometer and an Agilent 2100 Bioanalyzer. RNA-seq analysis was performed for patients from the Cys247X family as triplicate biological repeats (3 different differentiations) in hiPSC-derived RPE cells (Control, Cys247X, and Cys247X-As) and iPSC-derived retinal organoids at D100 and D130 (Control, Cys247X, Cys247X-Iso, and Cys247X-As).

For RPE cells, RNA-seq libraries were constructed from 1 µg of total RNAs using the TruSeq Stranded mRNA Sample Prep (Illumina) and paired-end sequencing of 75 bases length was performed on a HiSeq 4000 system (Illumina). For retinal organoids, RNA-seq libraries were constructed from 1 µg of total RNAs using the NEB Next Ultra II Directional RNA (New England Biolabs) and paired-end sequencing of 100 bases length was performed on a Novaseq system (Illumina). Image analysis and base calling are performed using the Illumina Real-Time Analysis (3.4.4) software with default parameters. Pass-filtered reads were mapped using STAR 2.7.3a^63^ and aligned to Ensembl genome assembly GRCh38 (release 98). This annotation includes cDNAs, miRNAs, long non-coding RNAs, pseudogenes, and gene predictions.

For differential expression analysis, a count table of the gene features was obtained using FeatureCounts^64^. For gene level analysis, EdgeR was used for normalization, differential expression analysis and to compute TPM (Transcripts Per Million) values^65^. A TPM filtering cutoff of five in at least one of the samples was applied.

Alternate splicing analysis was performed using rMATS^66^, allowing the analysis of differential exon usage for distinct types of events (skipped exons, alternative 5′ and 3′ splice sites, retained introns, and mutually exclusive exons). For each comparison being made, sorted BAM files produced by STAR (two-pass) were used to run rMATS with default settings. The identified splicing changes were filtered out using a minimum count of 2 for at least one sample, a false discovery rate (FDR) of <0.05, and a change in inclusion-level difference of more than 10%.

Comprehensive gene list analysis, enriched biological pathways, gene annotation from differential expression, and alternate splicing analysis were based on the Gene Ontology classification system using Metascape^67^. R packages were used for data mining, including GOPlot for pathway data graphical representation^68^.

### AAV production

To construct the pAAV-CAG-hPRPF31 rescue vector, the human *PRPF31* coding sequence was obtained from NCBI, gene fragments were synthesized (Integrated DNA Technologies) and cloned into a pAAV-CAG backbone. AAV7m8 vectors carrying human *PRPF3*1 cDNA or GFP were produced in 293AAV cells (Cell Biolabs) using a triple transfection method^69^. Recombinant AAVs were purified by iodixanol gradient ultracentrifugation, buffer exchanged, and concentrated with Amicon Ultra-15 Centrifugal Filter Units (#UFC8100) in PBS and titered by quantitative PCR relative to a standard curve using ITR-binding primers^70^.

### Infection of retinal organoids with AAV

Retinal organoids at D85 were placed one per well in a 96-well plate containing 50 μl of ProB27 medium with 10% FBS and 2 mM of Glutamax, and were transduced once at 4 × 10^9^ vg per organoid by direct addition in the culture medium. AAV used presented an engineered capsid, AAV2-7m8^54^, and carried the human *PRPF31* cDNA under the control of the CAG promoter. One day post infection, 50 μl of fresh medium was added per well. As control, infections with AAV2-7m8-CAG-GFP were carried out in the same manner as mentioned above. All transductions were performed in sextuplicates, and all data presented are representative of five independent experiments.

### Statistical analysis

*n* corresponds to the number of organoids or images from each independent differentiation; *N* indicates the number of independent experiments performed (e.g., the number of independent retinal differentiations). Statistical analyses represent the mean of at least three independent experiments. Data were averaged and expressed with means ± SD. Statistical analysis was performed using Prism 7 (GraphPad Software) with appropriate statistical tests, one-way ANOVA, or Kruskal–Wallis test, followed when necessary by multiple comparison tests such as Dunn’s or Tukey’s test. Values of *P* < 0.05 were considered statistically significant.

### Reporting summary

Further information on research design is available in the Nature Research Reporting Summary linked to this article.

## Supplementary information

Supplementary Figures

Dataset 1, Dataset 2, Dataset 3, Dataset 4, Dataset 5, Dataset 6, Dataset 7

REPORTING SUMMARY

## Data Availability

The raw RNA sequencing data are deposited in the Gene Expression Omnibus (GEO) database under accession code GSE206529. The remaining main data are available within the article or supplementary Information (figures and data files). Additional data inquiries could be addressed to the corresponding author (olivier.goureau@inserm.fr).

## References

[1] Hartong DT, Berson EL, Dryja TP (2006). Retinitis pigmentosa. Lancet.

[2] Verbakel SK (2018). Non-syndromic retinitis pigmentosa. Prog. Retin. Eye Res..

[3] Daiger SP, Sullivan LS, Bowne SJ (2013). Genes and mutations causing retinitis pigmentosa. Clin. Genet..

[4] Pan X (2014). Mutation analysis of pre-mRNA splicing genes in Chinese families with retinitis pigmentosa. Mol. Vis..

[5] Saini S, Robinson PN, Singh JR, Vanita V (2012). A novel 7 bp deletion in PRPF31 associated with autosomal dominant retinitis pigmentosa with incomplete penetrance in an Indian family. Exp. Eye Res..

[6] Sullivan LS (2013). Prevalence of mutations in eyeGENE probands with a diagnosis of autosomal dominant retinitis pigmentosa. Investig. Ophthalmol. Vis. Sci..

[7] Waseem NH (2007). Mutations in the gene coding for the Pre-mRNA splicing factor, PRPF31, in patients with autosomal dominant retinitis pigmentosa. Investig. Ophthalmol. Vis. Sci..

[8] Sato H (2005). Mutations in the pre-mRNA splicing gene, PRPF31, in Japanese families with autosomal dominant retinitis pigmentosa. Am. J. Ophthalmol..

[9] Martínez-Gimeno M (2003). Mutations in the pre-mRNA splicing-factor genes PRPF3, PRPF8, and PRPF31 in Spanish families with autosomal dominant retinitis pigmentosa. Investig. Ophthalmol. Vis. Sci..

[10] Audo I (2010). Prevalence and novelty of PRPF31 mutations in French autosomal dominant rod-cone dystrophy patients and a review of published reports. BMC Med. Genet..

[11] Utz VM, Beight CD, Marino MJ, Hagstrom SA, Traboulsi EI (2013). Autosomal dominant retinitis pigmentosa secondary to Pre-mRNA splicing-factor gene PRPF31 (RP11): review of disease mechanism and report of a family with a novel 3-base pair insertion. Ophthalmic Genet..

[12] Rose AM, Bhattacharya SS (2016). Variant haploinsufficiency and phenotypic non-penetrance in PRPF31 -associated retinitis pigmentosa. Clin. Genet..

[13] Wheway G, Douglas A, Baralle D, Guillot E (2020). Mutation spectrum of PRPF31, genotype-phenotype correlation in retinitis pigmentosa, and opportunities for therapy. Exp. Eye Res..

[14] Frio TR, Civic N, Ransijn A, Beckmann JS, Rivolta C (2008). Two trans-acting eQTLs modulate the penetrance of PRPF31 mutations. Hum. Mol. Genet..

[15] Frio TR (2009). A single-base substitution within an intronic repetitive element causes dominant retinitis pigmentosa with reduced penetrance. Hum. Mutat..

[16] Rivolta C (2006). Variation in retinitis pigmentosa-11 (PRPF31 or RP11) gene expression between symptomatic and asymptomatic patients with dominant RP11 mutations. Hum. Mutat..

[17] Rio Frio T (2008). Premature termination codons in PRPF31 cause retinitis pigmentosa via haploinsufficiency due to nonsense-mediated mRNA decay. J. Clin. Invest..

[18] Venturini G, Rose AM, Shah AZ, Bhattacharya SS, Rivolta C (2012). CNOT3 is a modifier of PRPF31 mutations in retinitis pigmentosa with incomplete penetrance. PLoS Genet..

[19] Rose AM (2014). Dominant PRPF31 mutations are hypostatic to a recessive CNOT3 polymorphism in retinitis pigmentosa: a novel phenomenon of “linked trans -acting epistasis”. Ann. Hum. Genet..

[20] Vithana EN (2001). A human homolog of yeast pre-mRNA splicing gene, PRP31, underlies autosomal dominant retinitis pigmentosa on chromosome 19q13.4 (RP11). Mol. Cell.

[21] Cao H (2011). Temporal and tissue specific regulation of RP-associated splicing factor genes PRPF3, PRPF31 and PRPC8—implications in the pathogenesis of RP. PLoS One.

[22] Tanackovic G (2011). PRPF mutations are associated with generalized defects in spliceosome formation and pre-mRNA splicing in patients with retinitis pigmentosa. Hum. Mol. Genet..

[23] Graziotto JJ (2011). Three gene-targeted mouse models of RNA splicing factor RP show late-onset RPE and retinal degeneration. Investig. Ophthalmol. Vis. Sci..

[24] Farkas MH (2014). Mutations in pre-mRNA processing factors 3, 8, and 31 cause dysfunction of the retinal pigment epithelium. Am. J. Pathol..

[25] Meyer JS (2011). Optic vesicle-like structures derived from human pluripotent stem cells facilitate a customized approach to retinal disease treatment. Stem Cells.

[26] Reichman S (2014). From confluent human iPS cells to self-forming neural retina and retinal pigmented epithelium. Proc. Natl Acad. Sci..

[27] Zhong X (2014). Generation of three-dimensional retinal tissue with functional photoreceptors from human iPSCs. Nat. Commun..

[28] Kuwahara A (2015). Generation of a ciliary margin-like stem cell niche from self-organizing human retinal tissue. Nat. Commun..

[29] Buskin A (2018). Disrupted alternative splicing for genes implicated in splicing and ciliogenesis causes PRPF31 retinitis pigmentosa. Nat. Commun..

[30] Brydon EM (2019). AAV-mediated gene augmentation therapy restores critical functions in mutant iPSC-derived PRPF31+/- cells. Mol. Ther. Methods Clin. Dev..

[31] Azizzadeh Pormehr, L., Ahmadian, S., Daftarian, N., Mousavi, S. A. & Shafiezadeh, M. PRPF31 reduction causes mis-splicing of the phototransduction genes in human organotypic retinal culture. *Eur. J. Hum. Genet*. **28**, 491–498 (2020). 10.1038/s41431-019-0531-1 PMC7080750 31654038

[32] Wheway G (2015). An siRNA-based functional genomics screen for the identification of regulators of ciliogenesis and ciliopathy genes. Nat. Cell Biol..

[33] Nazlamova L (2021). A CRISPR and high-content imaging assay compliant with ACMG/AMP guidelines for clinical variant interpretation in ciliopathies. Hum. Genet..

[34] Reichman S (2017). Generation of storable retinal organoids and retinal pigmented epithelium from adherent human iPS cells in xeno-free and feeder-free conditions. Stem Cells.

[35] Slembrouck-Brec A (2019). Reprogramming of adult retinal müller glial cells into human-induced pluripotent stem cells as an efficient source of retinal cells. Stem Cells Int..

[36] Vithana EN (2003). Expression of PRPF31 mRNA in patients with autosomal dominant retinitis pigmentosa: a molecular clue for incomplete penetrance?. Investig. Ophthalmol. Vis. Sci..

[37] Terray A (2017). Establishment of an induced pluripotent stem (iPS) cell line from dermal fibroblasts of an asymptomatic patient with dominant PRPF31 mutation. Stem Cell Res..

[38] Khabou, H. et al. Noninvasive gene delivery to foveal cones for vision restoration. *JCI Insight* **3**, e96029 (2018). 10.1172/jci.insight.96029 PMC5821199 29367457

[39] Garita-Hernandez M (2020). AAV-mediated gene delivery to 3d retinal organoids derived from human induced pluripotent stem cells. Int. J. Mol. Sci..

[40] Naylor A, Hopkins A, Hudson N, Campbell M (2019). Tight junctions of the outer blood retina barrier. Int. J. Mol. Sci..

[41] Wang S-B (2019). Disease-associated mutations of claudin-19 disrupt retinal neurogenesis and visual function. Commun. Biol..

[42] Kannan R, Sreekumar PG, Hinton DR (2012). Novel roles for α-crystallins in retinal function and disease. Prog. Retin. Eye Res..

[43] Lakkaraju A (2020). The cell biology of the retinal pigment epithelium. Prog. Retin. Eye Res..

[44] Rieu Q (2022). Pleiotropic roles of scavenger receptors in circadian retinal phagocytosis: a new function for lysosomal SR-B2/LIMP-2 at the RPE cell surface. Int. J. Mol. Sci..

[45] Gerding WM (2011). Ccdc66 null mutation causes retinal degeneration and dysfunction. Hum. Mol. Genet..

[46] Stingl K (2017). CDHR1 mutations in retinal dystrophies. Sci. Rep..

[47] Yang C (2021). Pre-mRNA processing factors and retinitis pigmentosa: RNA splicing and beyond. Front. Cell Dev. Biol..

[48] Arzalluz-Luque Á (2021). Mutant PRPF8 causes widespread splicing changes in spliceosome components in retinitis pigmentosa patient iPSC-derived RPE cells. Front. Neurosci..

[49] Deng W-L (2018). Gene correction reverses ciliopathy and photoreceptor loss in iPSC-derived retinal organoids from retinitis pigmentosa patients. Stem Cell Rep..

[50] Lane A (2020). Modeling and rescue of RP2 retinitis pigmentosa using iPSC-derived retinal organoids. Stem Cell Rep..

[51] Linder B (2011). Systemic splicing factor deficiency causes tissue-specific defects: a zebrafish model for retinitis pigmentosa†. Hum. Mol. Genet..

[52] Valdés-Sánchez L (2020). Retinal pigment epithelium degeneration caused by aggregation of PRPF31 and the role of HSP70 family of proteins. Mol. Med..

[53] Deery EC (2002). Disease mechanism for retinitis pigmentosa (RP11) caused by mutations in the splicing factor gene PRPF31. Hum. Mol. Genet..

[54] Dalkara D (2013). In vivo-directed evolution of a new adeno-associated virus for therapeutic outer retinal gene delivery from the vitreous. Sci. Transl. Med..

[55] Tosca L (2015). Genomic instability of human embryonic stem cell lines using different passaging culture methods. Mol. Cytogenet..

[56] Hockemeyer D (2009). Efficient targeting of expressed and silent genes in human ESCs and iPSCs using zinc-finger nucleases. Nat. Biotechnol..

[57] Cong L (2013). Multiplex genome engineering using CRISPR/cas systems. Science.

[58] Ran FA (2013). Genome engineering using the CRISPR-Cas9 system. Nat. Protoc..

[59] Gagliardi G (2018). Characterization and transplantation of CD73-positive photoreceptors isolated from human iPSC-derived retinal organoids. Stem Cell Rep..

[60] Legland D, Arganda-Carreras I, Andrey P (2016). MorphoLibJ: integrated library and plugins for mathematical morphology with ImageJ. Bioinformatics.

[61] Ferreira, T., Miura, K., Chef, B, & Eglinger, J. Scripts: bar 1.1.6. *Zenodo*10.5281/zenodo.28838 (2015).

[62] Parinot, C., Rieu, Q., Chatagnon, J., Finnemann, S. C. & Nandrot, E. F. Large-scale purification of porcine or bovine photoreceptor outer segments for phagocytosis assays on retinal pigment epithelial cells. *J. Vis. Exp*. **94**, 52100 (2014). 10.3791/52100 PMC4396958 25548986

[63] Dobin A (2013). STAR: ultrafast universal RNA-seq aligner. Bioinformatics.

[64] Liao Y, Smyth GK, Shi W (2014). featureCounts: an efficient general purpose program for assigning sequence reads to genomic features. Bioinformatics.

[65] Robinson MD, McCarthy DJ, Smyth GK (2010). edgeR: a Bioconductor package for differential expression analysis of digital gene expression data. Bioinformatics.

[66] Shen S (2012). MATS: a Bayesian framework for flexible detection of differential alternative splicing from RNA-Seq data. Nucleic Acids Res..

[67] Zhou Y (2019). Metascape provides a biologist-oriented resource for the analysis of systems-level datasets. Nat. Commun..

[68] Walter W, Sánchez-Cabo F, Ricote M (2015). GOplot: an R package for visually combining expression data with functional analysis. Bioinformatics.

[69] Grieger JC, Choi VW, Samulski RJ (2006). Production and characterization of adeno-associated viral vectors. Nat. Protoc..

[70] Aurnhammer C (2012). Universal real-time PCR for the detection and quantification of adeno-associated virus serotype 2-derived inverted terminal repeat sequences. Hum. Gene Ther. Methods.

