# Supplementary material for: Modeling PRPF31 retinitis pigmentosa using retinal pigment epithelium and organoids combined with gene augmentation rescue
Source: NPJ Regen Med. 2022 Aug 16;7:39. doi: 10.1038/s41536-022-00235-6 (PMC9381579; doi:10.1038/s41536-022-00235-6)
Supplement: Supplementary file 1 — Supplementary Figures [file 41536_2022_235_MOESM1_ESM.pdf]

SUPPLEMENTARY FILES

Supplementary Figures

Supplementary Figure 1. Derivation and characterization of iPSCs from and RP patient carrying a *PRPF31* mutation and a non-carrier family-related member.

Supplementary Figure 2. Derivation and characterization of a *PRPF31*-mutated (Tyr90CysfsX21) human iPSC line.

Supplementary Figure 3. Generation and characterization of the isogenic control iPSC line by CRISPR/Cas9.

Supplementary Figure 4. Comparison of different cell shape parameters in iRPE cells derived from different PRPF31 patient iPSCs.

Supplementary Figure 5. Generation and characterization of *PRPF31*-mutated iPSCs expressing a supplementary copy of *PRPF31*.

Supplementary Figure 6. AAV-GFP efficiently transduce iPSC-derived retinal organoids.

Supplementary Figure 7. Heatmap of RPE markers expressed in iRPE cells.

Supplementary Figure 8. Selected clusters corresponding to the deregulated genes in iRPE cells derived from Cys247X patients (Heatmap representation).

Supplementary Figure 9. Selected clusters corresponding to the deregulated genes in D100 retinal organoids between Cys247X iPSCs and Cys247X-Isogenic corrected iPSCs (Heatmap representation).

Supplementary Figure 10. Identification of genes/pathways deregulated and alternate splicing in D130 retinal organoids derived from PRPF31 patient iPSCs.

Supplementary Figure 11. Selected clusters corresponding to the deregulated genes in in D130 retinal organoids.

Supplementary Datas

Supplementary Data 1. Off-target site sequencing analysis for gRNA used to correct the Cys247X mutation.

Supplementary Data 2. Differential gene expression in iRPE cells for Cys247X and Cys247X-asymptomatic as well as Control patients (Filtering: FC2, FDR $\leq$ 0.05, TMP $\geq$ 5).

Supplementary Data 3. Differential gene expression in iPSC-derived retinal organoids at D100 and D130 for Cys247X versus Cys247X-Isogenic, Cys247X-Asymptomatic and Control patients (Filtering: FC2, Pval $\leq$ 0.05, TMP $\geq$ 5).

Supplementary Data 4. Differential exon usage in iRPE cells of Cys247X versus Cys247X-asymptomatic and Control patients (Filtering: PSI $\geq$ 0.1, deltaPSI $\geq$ 0.1, FDR $\leq$ 0.05).

Supplementary Data 5. Differential exon usage in iPSC-derived retinal organoids at D100 and D130 of Cys247X versus Cys247X-Isogenic, Cys247X-Asymptomatic and Control patients (Filtering: PSI $\geq$ 0.1, deltaPSI $\geq$ 0.1, FDR $\leq$ 0.05).

Supplementary Data 6. Summary of antibodies used, Taqman primers, mutation screening primers, and CRISPR-Cas9 gRNA/ssODN/primers.

Supplementary Data 7. Uncropped blots from Figure 1g, 3e, 5a, 6e and 7e.

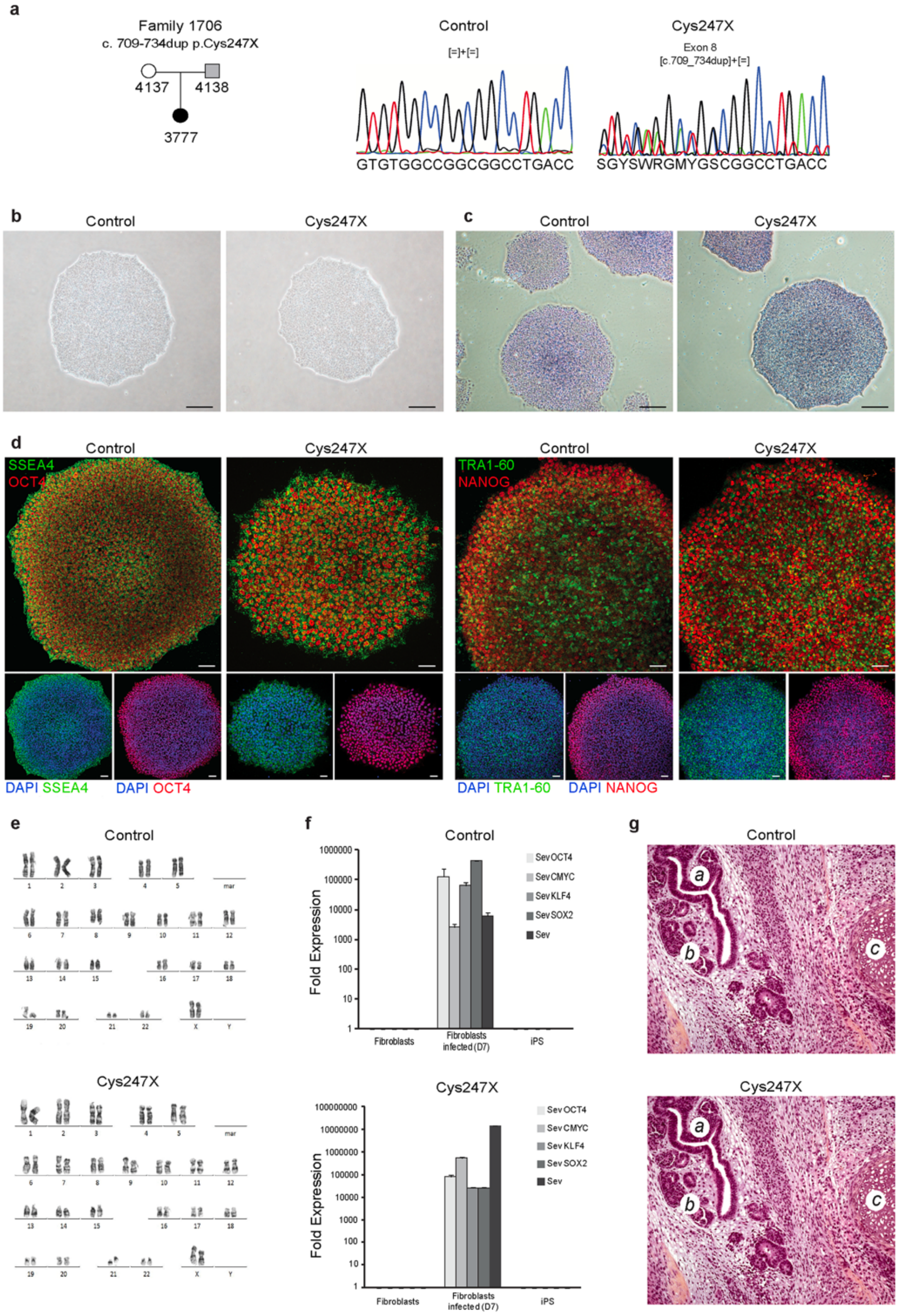

Supplementary Figure 1. Derivation and characterization of iPSCs from a RP patient carrying a *PRPF31* mutation and a non-carrier family-related member.

a. Pedigree of a *PRPF31* family and Sanger sequence trace of fibroblasts from heterozygous c709_734dup patient 3777 (Cys247X) and non-carrier patient 4137 (Control). **b.** Bright-field images of representative iPSC colonies derived from Control and Cys247X patients. Scale bar = 100 μm. **c.** Positive alkaline phosphatase staining of control and Cys247X iPS colonies. Scale bar = 100 μm. **d.** Immunofluorescence staining of pluripotency markers OCT4, SSEA4, NANOG and TRA1-60 for control and Cys247X iPSCs. Scale bars = 100 μm. **e.** Karyotype analysis of control and Cys247X iPSC lines. **f.** Verification by RT-qPCR of Sendai virus genome (*Sev*) and exogenous reprogramming factors clearance in control and Cys247X iPSCs after 10 passages. One-week infected-fibroblasts were used as a positive control and data are normalized to uninfected fibroblasts. **g.** Histological analysis of iPSC-generated teratomas in NSG mouse with (a) neural tube, (b) bone, and (c) cartilage.

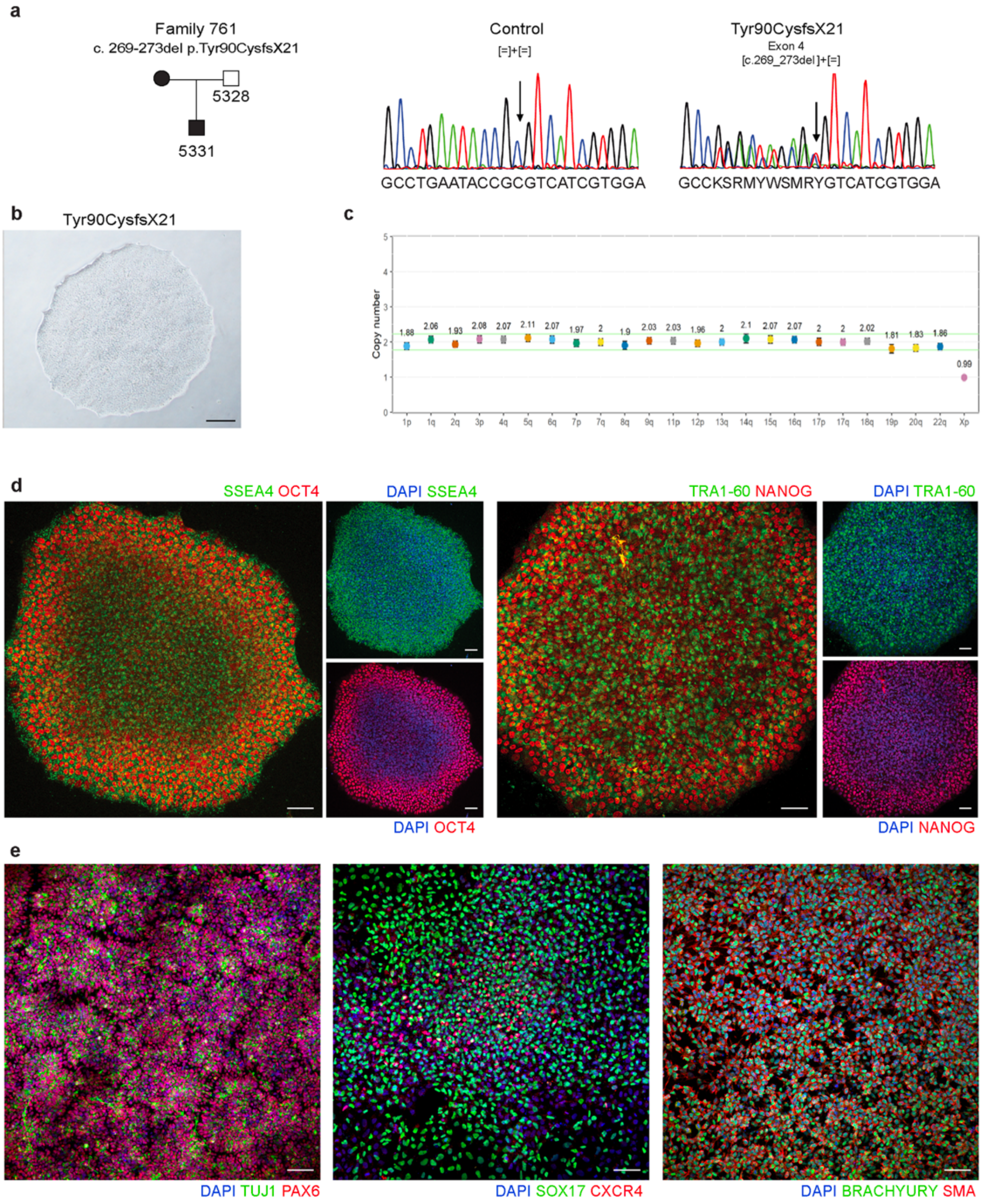

Supplementary Figure 2. Derivation and characterization of a *PRPF31*-mutated (Tyr90CysfsX21) iPSC line.

a. Pedigree of PRPF31 family and Sanger sequence trace of fibroblasts from heterozygous c269_273del patient 5331 (Tyr90CysfsX21). **b.** Bright-field image of a representative iPSC colony derived from Tyr90CysfsX21 patient. Scale bar = 100 µm. **c.** Genomic stability of Tyr90CysfsX21 iPSCs assessed by digital q-PCR analysis of the most common iPSC genetic abnormalities (iCS-digital^{TM} PSC test, Stem Genomics). **d.** Immunofluorescence staining of pluripotency markers OCT4, SSEA4, NANOG and TRA1-60 for Tyr90CysfsX21 iPSCs. Scale bars = 100 µm. **e.** Differentiation capacity of Tyr90CysfsX21 iPSCs assessed by immunofluorescence staining of the germ layers markers TUJ1 and PAX6 (ectoderm), SOX17 and CXCR4 (endoderm) and BRACHYURY and SMA (mesoderm). Nuclei were counterstained with DAPI. Scale bar = 100 µm.

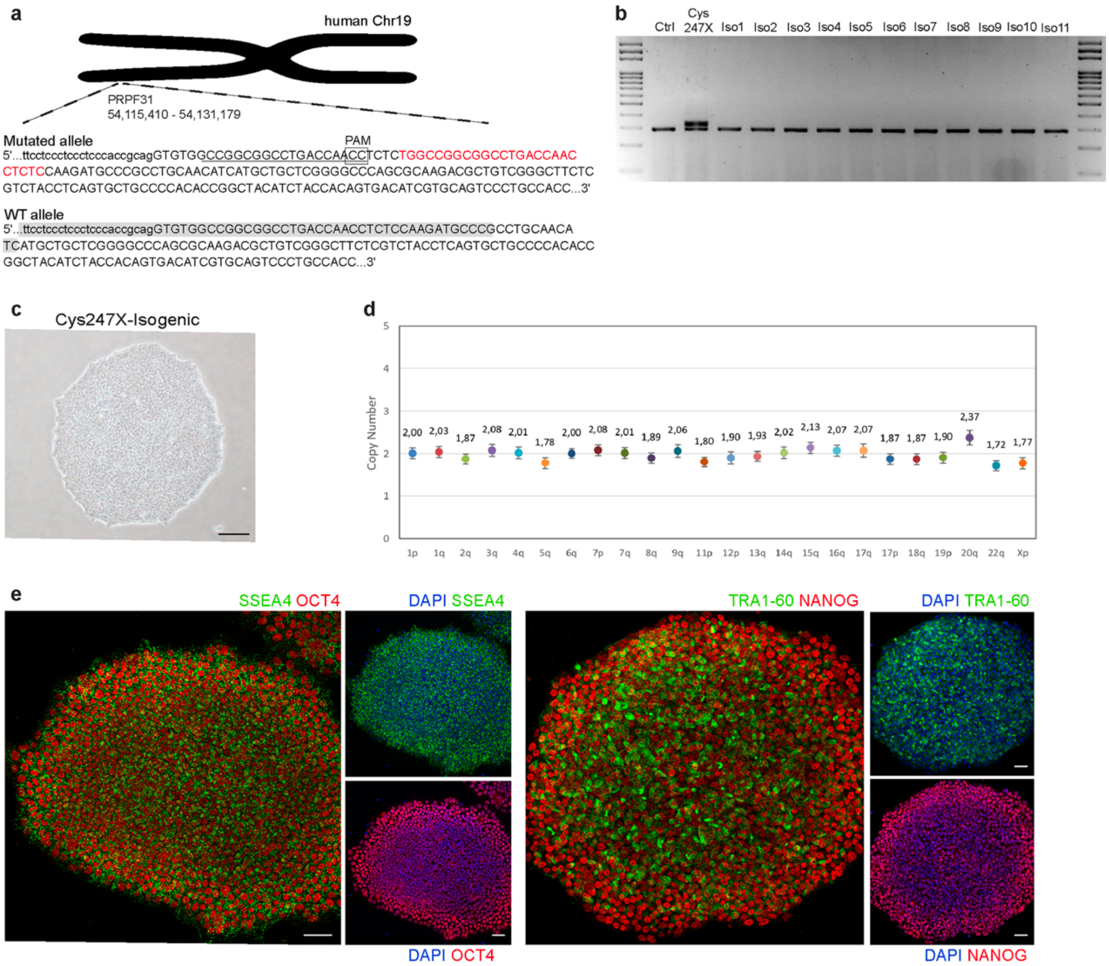

Supplementary Figure 3. Generation and characterization of the isogenic control iPSC line by CRISPR/Cas9.

a. Diagram showing the design of the gRNA (underlined) targeting specifically the mutated allele (upper sequence) with the 25-nucleotide duplication shown in red and the sequence of exogenous repair template ssODN highlighted in grey on the wild-type allele (lower sequence), used to correct the c709_734dup *PRPF31* mutation and therefore generate Cys247X-Isogenic iPSCs. **b.** Results of the PCR evaluating the correction of the mutated allele. Amplification of the Cys247X heterozygous mutant lead to the amplification of the WT allele and the mutated allele (25 nucleotides larger). Gel shows only one band, corresponding to the amplification of the WT allele, in clones Iso2 to 11, confirming the correction of the mutated allele. **c.** Bright-field images of a representative colony of Cys247X-Isogenic (Iso2) iPSCs. Scale bar = 100 µm. **d.** Genomic stability of Cys247X-Isogenic iPSCs (Iso2) assessed by digital q-PCR analysis of the most common iPSC genetic abnormalities (iCS-digital^{TM} PSC test, Stem Genomics). **e.** Pluripotency of Cys247X-Isogenic iPSCs (Iso2) assessed by immunofluorescence staining with markers OCT4, SSEA4, NANOG and TRA1-60. Nuclei were counterstained with DAPI. Scale bar = 100 µm.

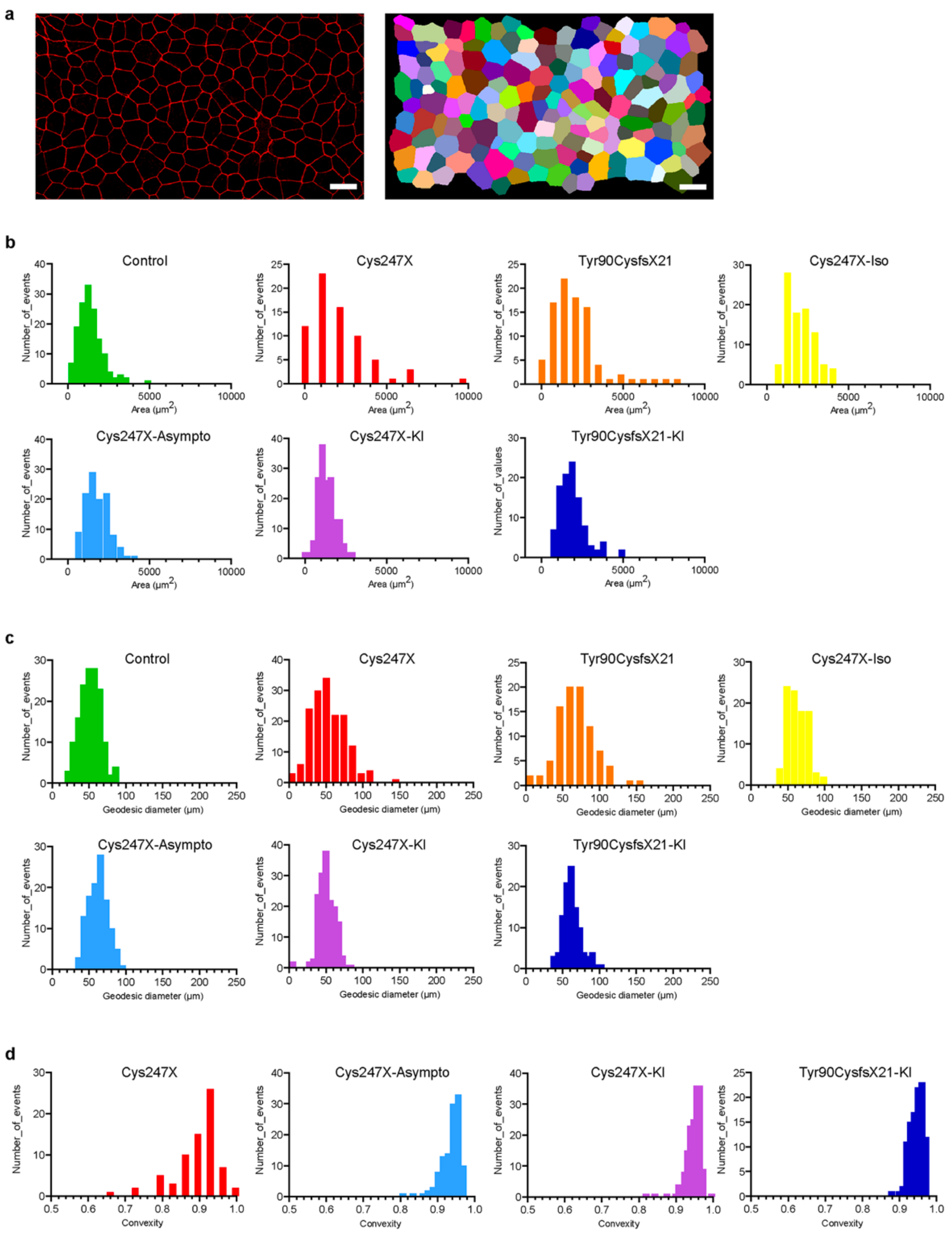

Supplementary Figure 4. Comparison of different cell shape parameters in iRPE cells derived from different *PRPF31* patient iPSCs.

a. Quantification of RPE cell area and shape parameters. ZO-1 signals (left panel) were filtered and binarized to apply Voronoi function, then colorized (Glasbey LUT) and MorpholibJ plugin was used to analyze cell shape parameters of interest by on detected individual cells (right panel). **b.** Distribution of iRPE cells based on the area (number of pixels) parameter. **c.** Distribution of iRPE cells based on the and Geodesic Diameter (largest geodesic distance between two points within an individual image) parameter. **d.** Distribution of iRPE cells based on the convexity (ratio convex perimeter/perimeter) parameter.

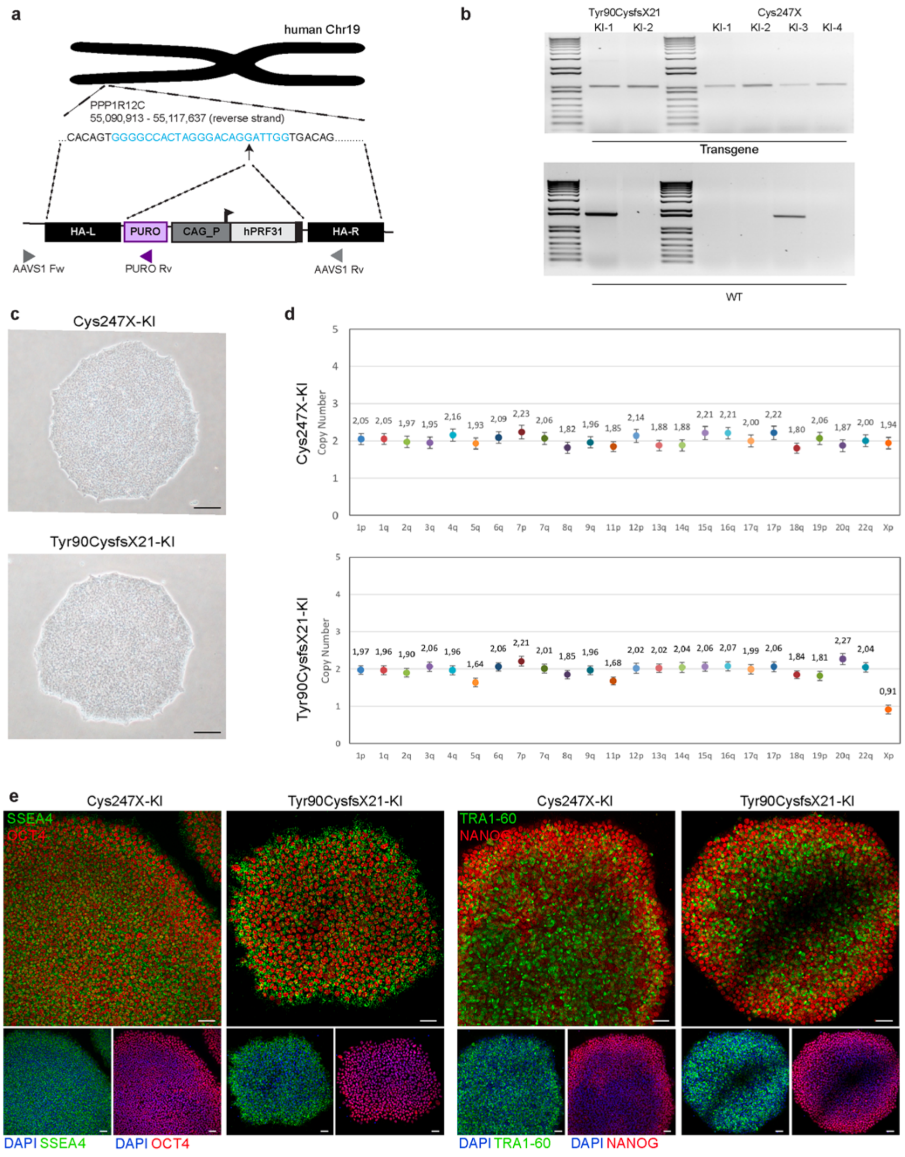

Supplementary Figure 5. Generation and characterization of *PRPF31*-mutated iPSCs expressing a supplementary copy of *PRPF31*.

a. Schematic representation of CRISPR/Cas9, gRNA and inserted cassette used for the generation of the AAVS1::CAG-P_PRPF31 iPSCs. Triangles on the schema indicate the homology region of the primers (AAVS1 Fw, PURO Rv, AAVS1 Rv) used to validate the insertion of *PRPF31*. **b.** Results of the PCR evaluating the integration of CAG-P_PRPF31 construct into AAVS1 site. Gel on the top shows the presence of the transgene in all puromycin-selected clones corresponding to the amplification product with AAVS1 Fw (upstream the integration site) and PURO Rv primers (within the cassette), as indicated in A. Gel on the bottom shows the result of the PCR with AAVS1 Fw and AAVS1 Rv primers (in the AAVS1 right homologous arm) amplifying exclusively the WT form of the AAVS1 locus allowing for distinction between homozygous (Tyr90CysfsX21 KI-2 and Cys247X KI-1, KI-2, KI-4) or heterozygous (Tyr90CysfsX21 KI-1 and Cys247X KI-3) integration. **c.** Bright-field images of a representative colony of Cys247X-KI (KI-3) and Tyr90CysfsX21-KI (KI-1) iPSCs. Scale bar = 100 µm. **d.** Genomic stability of Cys247X-KI (KI-3) and Tyr90CysfsX21-KI (KI-1) iPS cells assessed by digital q-PCR analysis of the most common iPSC genetic abnormalities (iCS-digital^{TM} PSC test, Stem Genomics). **e.** Pluripotency of Cys247X-KI (KI-3) and Tyr90CysfsX21-KI (KI-1) iPSCs assessed by immunofluorescence staining with markers OCT4, SSEA4, NANOG and TRA1-60. Nuclei were counterstained with DAPI. Scale bar = 100 µm.

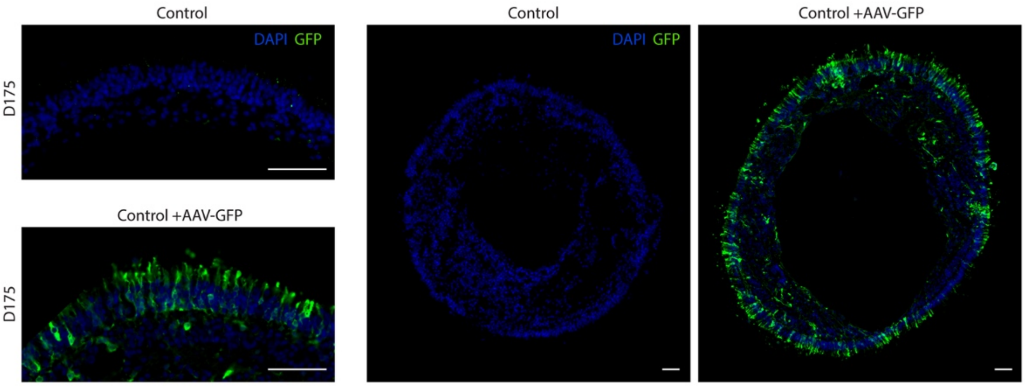

Supplementary Figure 6. AAV-GFP efficiently transduced iPSC-derived retinal organoids.
Immunofluorescence staining of cryosections from D175 retinal organoids not transduced (Control) or previously transduced at D85 with AAV-CAG-GFP (Control +AAV-GFP) using GFP antibody. Nuclei were counterstained with DAPI. Scale bar = 100 µm.

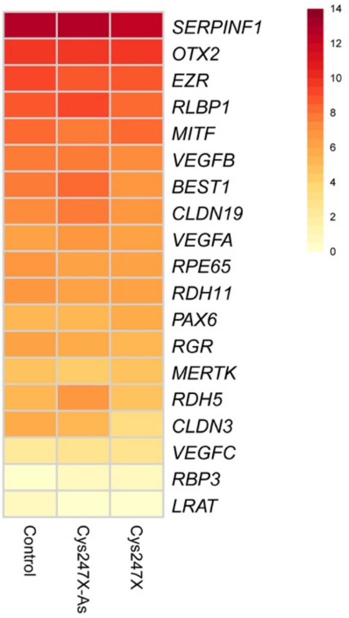

Supplementary Figure 7. Heatmap of RPE markers expressed in iRPE cells.
Heatmaps of a selection of RPE-expressed genes. Expression values correspond to the mean Log2(TPM) for each group. Cys247X-As = Cys247X-Asympto.

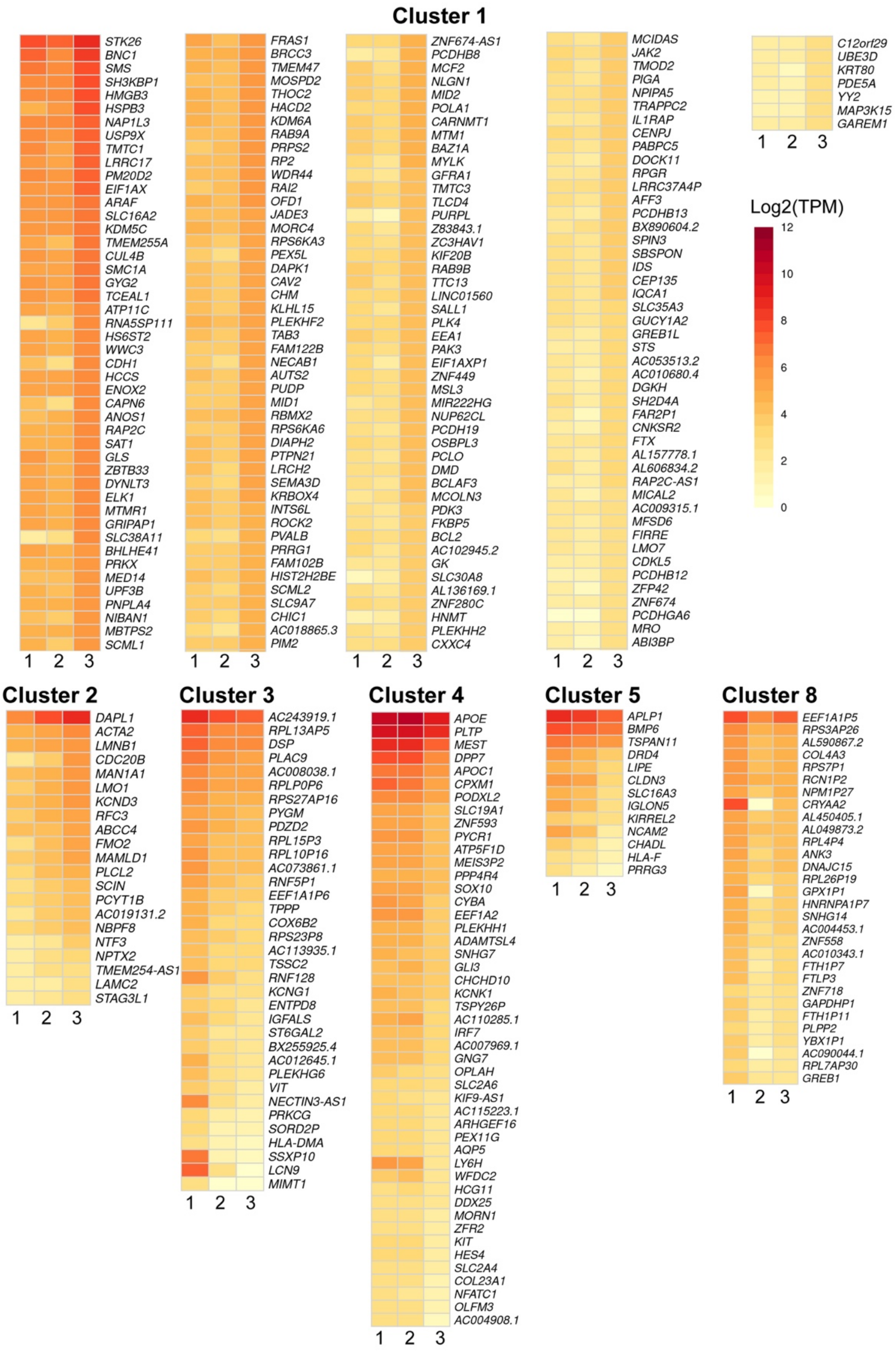

Supplementary Figure 8. Selected clusters corresponding to the deregulated genes in iRPE cells from Cys247X patients.

Heatmaps of the 6 selected clusters of interest corresponding to deregulated genes in Cys247X-derived iRPE cells. Expression values correspond to the mean Log2(TPM) for each group. Lane 1, Control; lane 2, Cys247X-Asympto; lane 3, Cys247X.

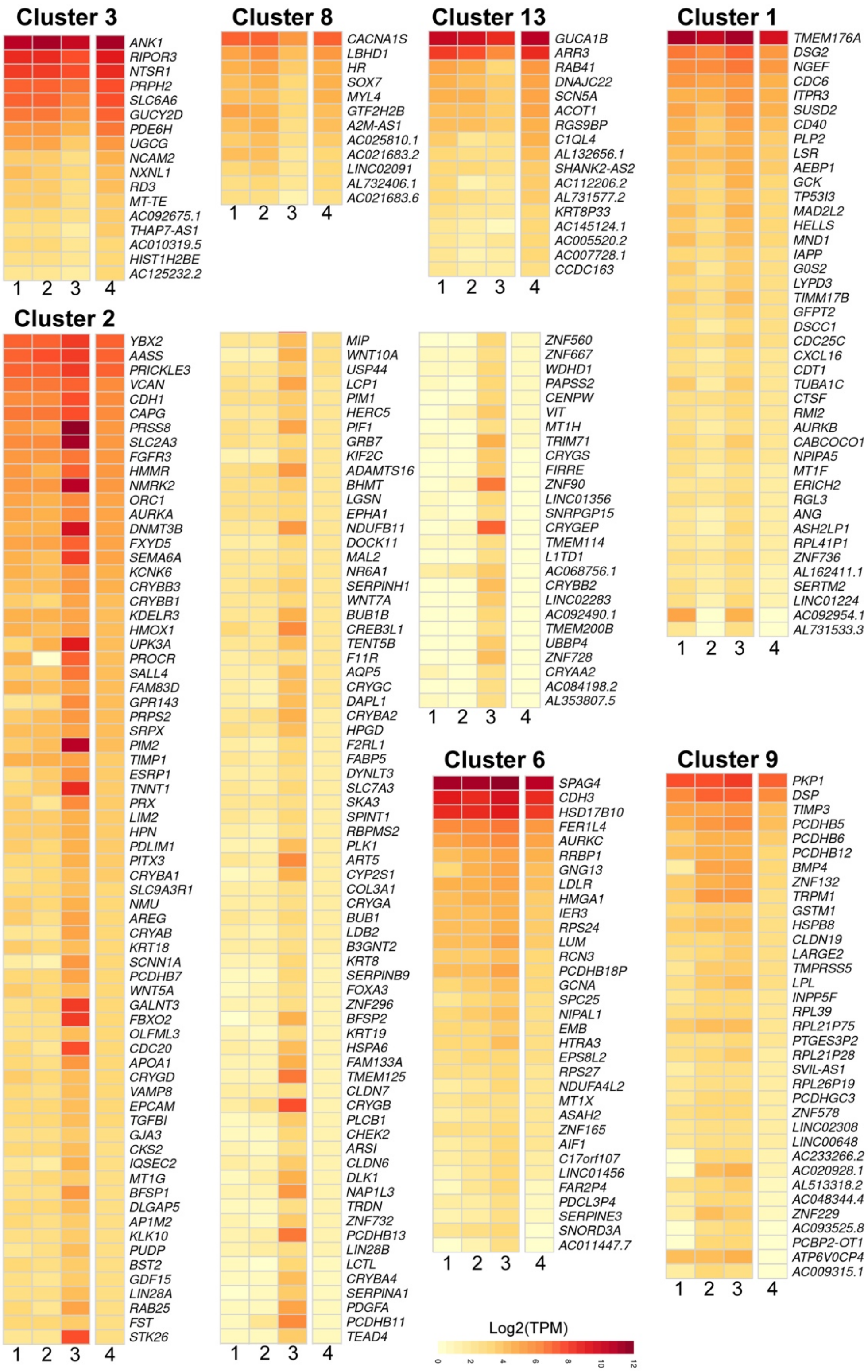

Supplementary Figure 9. Selected clusters corresponding to the deregulated genes in D100 retinal organoids between Cys247X iPSCs and Cys247X-Isogenic corrected iPSCs.

Heatmaps of the 7 selected clusters of interest corresponding to deregulated genes in Cys247X-Iso *vs* Cys247X -derived retinal organoids at D100. Expression values correspond to the mean Log2(TPM) for each group. Lane 1, Control; lane 2, Cys247X-Asympto; lane 3, Cys247X; lane 4, Cys247X-Iso.

a

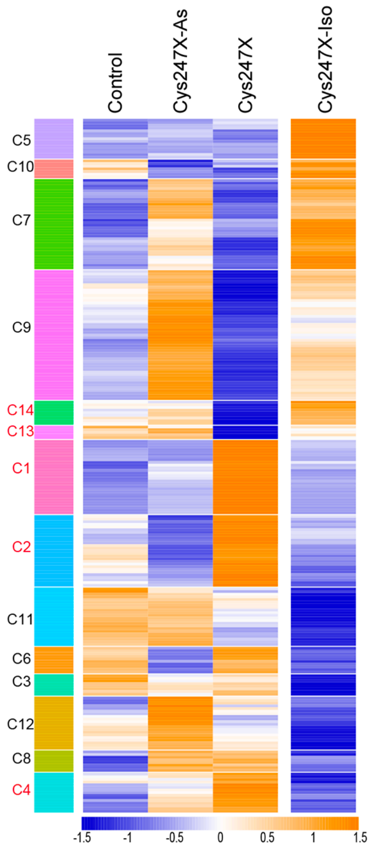

d

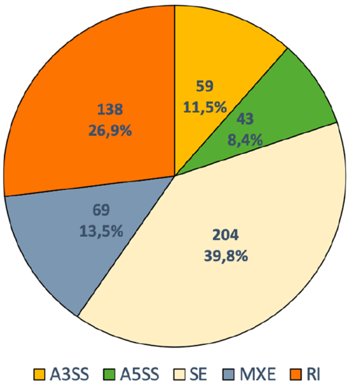

b

Category	ID	Term	Count	List Total	*pval*
BP	GO:0007601	visual perception	10	218	1.31E-10
BP	GO:0001654	eye development	11	384	2.14E-09
BP	GO:0098742	cell-cell adhesion via plasma-membrane adhesion molecules	8	279	3.88E-07
BP	GO:0030198	extracellular matrix organization	8	397	5.36E-06
BP	GO:0071774	response to fibroblast growth factor	4	153	5.32E-04
BP	GO:0021700	developmental maturation	5	287	6.73E-04
CC	GO:0031012	extracellular matrix	8	569	7.02E-05
MF	GO:0005509	calcium ion binding	13	712	1.45E-08
MF	GO:0001228	DNA-binding transcription activator activity, RNA polymerase II-specific	8	516	3.54E-05
MF	GO:0019838	growth factor binding	3	137	4.56E-03

e

Category	ID	Term	adj_pval
BP	GO:0043484	regulation of RNA splicing	5.88E-11
BP	GO:0010639	negative regulation of organelle organization	5.66E-04
BP	GO:0022613	ribonucleoprotein complex biogenesis	9.20E-04
BP	GO:0032989	cellular component morphogenesis	2.19E-03
BP	GO:0030036	actin cytoskeleton organization	3.06E-03
BP	GO:0051028	mRNA transport	1.89E-02
CC	GO:1990904	ribonucleoprotein complex	2.04E-05
CC	GO:0030424	axon	7.21E-04
CC	GO:0005911	cell-cell junction	3.63E-03
CC	GO:0097733	photoreceptor cell cilium	1.81E-02
CORUM	CORUM:351	Spliceosome	3.74E-04
MF	GO:0008017	microtubule binding	2.25E-04
MF	GO:0005516	calmodulin binding	5.87E-03
MF	GO:0008289	lipid binding	6.11E-03
MF	GO:0019904	protein domain specific binding	6.23E-03
MF	GO:0003712	transcription coregulator activity	9.45E-03
MF	GO:0016301	kinase activity	1.26E-02

c

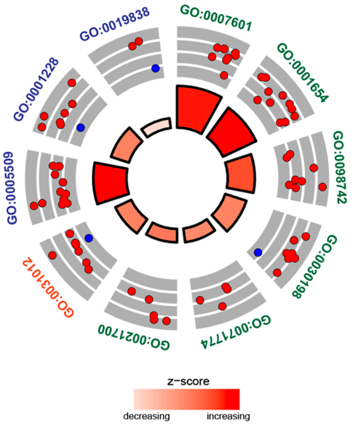

f

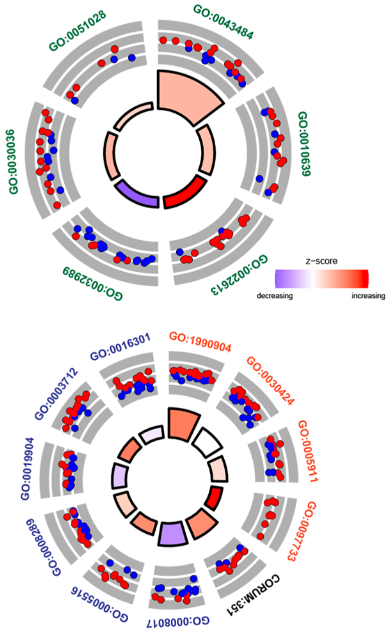

Supplementary Figure 10. Identification of genes and pathways deregulated and alternate splicing in D130 retinal organoids derived from *PRPF31* patient iPSCs.

a. Hierarchical clustering analysis of the DEGs between Cys247X-Iso and Cys247X with genes up- or down-regulated by a factor ≥2 with Pval ≤0.05 in D130 retinal organoids. The *z-score* was derived from the average of the replicates for each experimental group. Blue: low expression; orange: high expression. The top row represents the 14 clusters. Selected clusters used for further pathway analysis are in red. **b.** Table of the over-represented GO pathways of interest identified with Metascape using the DEGs from the selected clusters. The different categories of pathways are BP, Biological Process; CC, Cell Compartment; MF, Molecular Function. **c.** Circular visualization of selected GO enriched pathways. Down-regulated genes (blue dots) and up-regulated genes (red dots) within each GO pathway of interest are plotted based on logFC. *Z-score* bars indicate if an entire biological process is more likely to be increased or decreased based on the genes it comprises. Green, Biological Process; orange, Cell Compartment; blue, Molecular Function. **d.** Pie chart from rMATs analysis showing the proportion of splicing events in D130 organoids. A5SS and A3SS, alternative 5' and 3' splice sites; SE, skipped exons; RI, retained introns; MXE, mutually exclusive exons. **e.** Table showing pathway identity number (ID), associated pathway name (Term), gene count from pathways analysis and the adjusted *P-value* (adj_pval). The different categories of pathways are BP, Biological Process; CC, Cell Compartment; MF, Molecular Function. **f.** Circular visualization of selected GO enriched pathways from the identified 600 genes with alternative splicing events (total of 1049 splicing events) between Cys247X-Iso and Cys247X retinal organoids at D100. Down-regulated genes (blue dots) and up-regulated genes (red dots) within each GO pathway are plotted based on logFC. *Z-score* bars indicate if an entire biological process is more likely to be increased or decreased based on the genes it comprises. Green, Biological Process; orange, Cell Compartment; blue, Molecular Function; dark, CORUM.

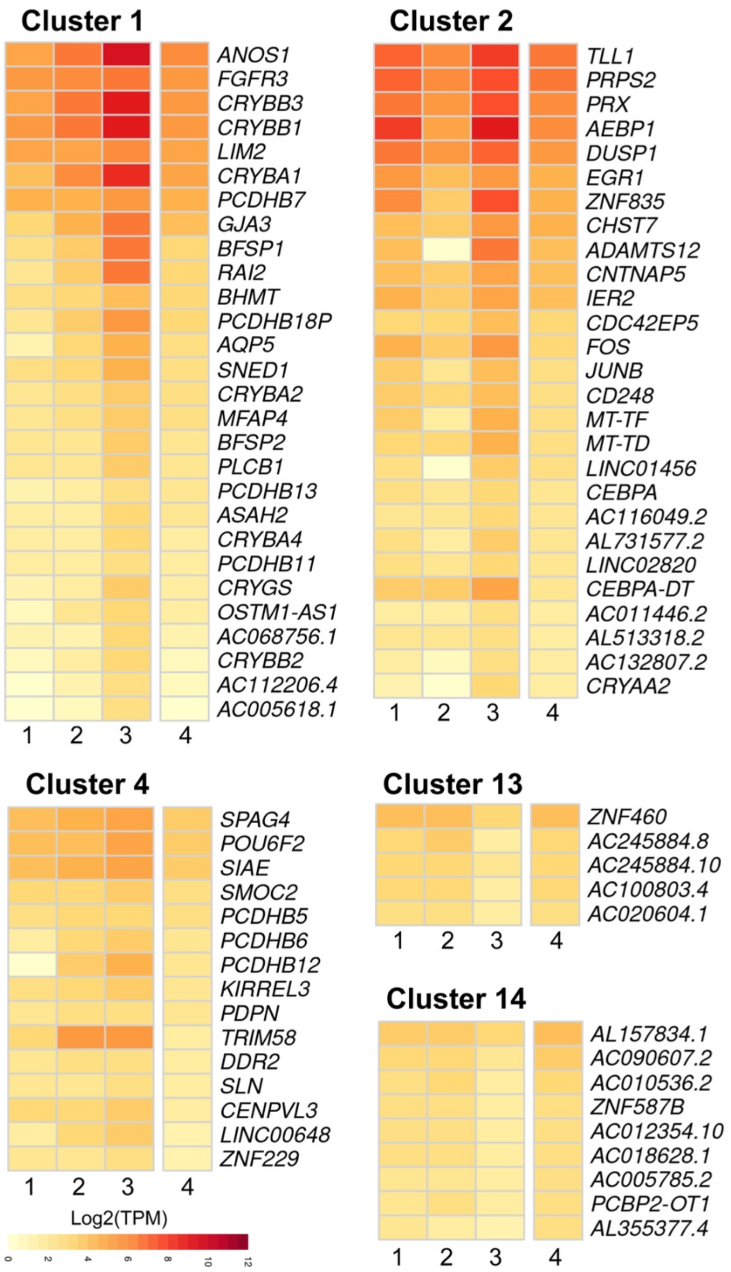

Supplementary Figure 11. Selected clusters corresponding to the deregulated genes in D130 retinal organoids.

Heatmaps of the 5 selected clusters of interest corresponding to deregulated genes in Cys247X-Iso v*s* Cys247X -derived retinal organoids at D130. Expression values correspond to the mean Log2(TPM) for each group. Lane 1, Control; lane 2, Cys247X-Asympto; lane 3, Cys247X; lane 4, Cys247X-Iso.
