# Supplementary material for: Modeling PRPF31 retinitis pigmentosa using retinal pigment epithelium and organoids combined with gene augmentation rescue
Source: NPJ Regen Med. 2022 Aug 16;7:39. doi: 10.1038/s41536-022-00235-6 (PMC9381579; doi:10.1038/s41536-022-00235-6)
Supplement: Supplementary file 3 — REPORTING SUMMARY [file 41536_2022_235_MOESM3_ESM.pdf]

nature portfolio

Corresponding author(s): GOUREAU Olivier

Last updated by author(s): Jun 21, 2022

Reporting Summary

Nature Portfolio wishes to improve the reproducibility of the work that we publish. This form provides structure for consistency and transparency in reporting. For further information on Nature Portfolio policies, see our Editorial Policies and the Editorial Policy Checklist.

Statistics

For all statistical analyses, confirm that the following items are present in the figure legend, table legend, main text, or Methods section.

n/a	Confirmed	
☐	☒	The exact sample size (*n*) for each experimental group/condition, given as a discrete number and unit of measurement
☐	☒	A statement on whether measurements were taken from distinct samples or whether the same sample was measured repeatedly
☐	☒	The statistical test(s) used AND whether they are one- or two-sided<br>*Only common tests should be described solely by name; describe more complex techniques in the Methods section.*
☒	☐	A description of all covariates tested
☐	☒	A description of any assumptions or corrections, such as tests of normality and adjustment for multiple comparisons
☐	☒	A full description of the statistical parameters including central tendency (e.g. means) or other basic estimates (e.g. regression coefficient) AND variation (e.g. standard deviation) or associated estimates of uncertainty (e.g. confidence intervals)
☒	☐	For null hypothesis testing, the test statistic (e.g. *F*, *t*, *r*) with confidence intervals, effect sizes, degrees of freedom and *P* value noted<br>*Give P values as exact values whenever suitable.*
☒	☐	For Bayesian analysis, information on the choice of priors and Markov chain Monte Carlo settings
☒	☐	For hierarchical and complex designs, identification of the appropriate level for tests and full reporting of outcomes
☒	☐	Estimates of effect sizes (e.g. Cohen's *d*, Pearson's *r*), indicating how they were calculated

Our web collection on statistics for biologists contains articles on many of the points above.

Software and code

Policy information about availability of computer code

Data collection	No software was used to collect data.
Data analysis	CRISPOR tool website (http://crispor.tefor.net)<br>FIJI / Image J open source software (NIH, USA)<br>Prism 7 software (GraphPad)<br>MorpholibJ: Legland et al. Bioinformatics 32, 3532–3534 (2016)<br>BAR: Ferreira et al. J. Scripts: Bar 1.1.6. Zenodo (2015)<br>STAR 2.7.3a: Dobin A. et al. Bioinformatics 29, 15–21 (2013)<br>FeatureCounts: Liao et al. Bioinformatics 30, 923–930 (2014)<br>EdgeR: Robinson et al. Bioinformatics 26, 139–140 (2010)<br>rMATS: Shen et al. Nucleic Acids Res. 40, e61–e61 (2012)<br>Metascape : Zhou et al. Nat. Commun. 10, 1523 (2019)<br>R packages (GOPlot): Walter et al. Bioinformatics 31, 2912–2914 (2015)

For manuscripts utilizing custom algorithms or software that are central to the research but not yet described in published literature, software must be made available to editors and reviewers. We strongly encourage code deposition in a community repository (e.g. GitHub). See the Nature Portfolio guidelines for submitting code & software for further information.

Data

Policy information about availability of data

All manuscripts must include a data availability statement. This statement should provide the following information, where applicable:

- Accession codes, unique identifiers, or web links for publicly available datasets
- A description of any restrictions on data availability
- For clinical datasets or third party data, please ensure that the statement adheres to our policy

The main data supporting the findings of this study are available within the paper and its supplementary information files: The source data underlying RNASeq analysis in Figures 8B, 9B,10A and B, 10B and in Supplementary Figures 10A and D are provided as a Supplementary Data 2, 3, 4 and 5 and the Uncropped blots from Figure 1g, 3e, 5a, 6e and 7e are provided in Supplementary Data 7)
The raw RNA sequencing data are deposited in the GEO database under accession code GSE206529.
Extra data are available from the corresponding author upon reasonable request (olivier.goureau@inserm.fr).

Field-specific reporting

Please select the one below that is the best fit for your research. If you are not sure, read the appropriate sections before making your selection.

☒ Life sciences ☐ Behavioural & social sciences ☐ Ecological, evolutionary & environmental sciences

For a reference copy of the document with all sections, see nature.com/documents/nr-reporting-summary-flat.pdf

Life sciences study design

All studies must disclose on these points even when the disclosure is negative.

Sample size	There was no sample-size calculation performed. We used other similar studies on iPSC derivatives as a guide in choosing the sample sizes (minimum of three biological samples from three different experiments)
Data exclusions	No data were excluded
Replication	The reproducibility of the experimental findings was confirmed within our laboratory by replication of three parallel biological samples from three different groups of differentiation experiments for each iPS cell line.
Randomization	This is not relevant to the present study since the experimental and control groups were determined according to the iPS cells derived from patients and healthy individuals, respectively.
Blinding	For the immunohistochemistry experiments of iPSC-derived RPE and organoids, investigators were blinded to the iPS cell line. Blinding was not relevant to other assays since there is no potential bias.

Reporting for specific materials, systems and methods

We require information from authors about some types of materials, experimental systems and methods used in many studies. Here, indicate whether each material, system or method listed is relevant to your study. If you are not sure if a list item applies to your research, read the appropriate section before selecting a response.

Materials & experimental systems

n/a	Involved in the study	
☐	☒	Antibodies
☐	☒	Eukaryotic cell lines
☒	☐	Palaeontology and archaeology
☒	☐	Animals and other organisms
☒	☐	Human research participants
☒	☐	Clinical data
☒	☐	Dual use research of concern

Methods

n/a	Involved in the study	
☒	☐	ChIP-seq
☒	☐	Flow cytometry
☒	☐	MRI-based neuroimaging

Antibodies

Antibodies used	All the antibodies used are listed in Supplementary Data 6
Validation	The CONE ARRESTINE (hCAR) antibody was provided by Cheryl Craft (University of Southern California) and validated in various papers, including our own (Slembrouck-Brec et al. 2019: doi: 10.1155/2019/7858796).

All other antibodies listed below are commercial antibodies tested by the respective manufacturers for IF or WB and by our group in previous papers (Reichman et al. 2107 doi:10.1002/stem.2586; Slembrouck-Brec et al. 2019: doi: 10.1155/2019/7858796) :
Bestrophin 1: Novus Biologicals (NB300-164)
Cleaved CASP3: Cell Signaling Tech. (#9661)
CRALBP : Abcam (ab15051)
Ezrin: Sigma-Aldrich (E8897)
GAPDH: Abcam (ab9485)
GFP: AVES lab. (GFP-1020)
MERTK: Abcam (ab52968)
NANOG: Cell Signaling Tech. (#99399)
NRL: R&D systems (AF2945)
OCT4: Cell Signaling Tech. (#94310)
PRPF31: Abcam (ab188577)
Protein Kinase C alpha: Santa-Cruz (sc-208)
Rhodopsin: Merck/Millipore (MABN15)
SSEA4: Cell Signaling Tech. (#43782)
TRA1-60: Cell Signaling Tech. (#61220)
VSX2 (CHX10): Santa Cruz (SC-21690)
ZO1: Thermo Fisher Scientific (61-7300)

Eukaryotic cell lines

Policy information about cell lines

Cell line source(s)	All experiments were carried out using iPS cell lines generated in this study, with the exception of the iPS cell line derived from an asymptomatic PRPF31 carrier, the full caraterisation of which has been previously published (Terray et al. 2014: doi: 10.1016/j.scr.2017.10.007).<br>All skin biopsies used in the study for fibroblasts isolation and iPS generation were obtained from patients who provided written informed consent to take part in the study under the approval by French regulatory agencies: CPP Ile de France (2012-A01333–40; P12-02) and the French Agency for the Safety of Health Products (ANSM) (B121362-32).
Authentication	Authentication of the iPS cell lines shown in Supplementary Figures 1 and 2 consisted of positive alkaline phosphatase staining, immunohistochemistry of pluripotency markers (SSEA4, OCT4, TRA1-60 and NANOG) and capacity for differentiation towards the three main germ layers markers either by teratoma formation in NSG mouse or by in vitro trilineage differentiation (ectoderm PAX6, TUJ1; mesoderm, BRACHYURY, SMA and endoderm, SOX17, CXCR4). The genomic integrity was evaluated by karyotype analysis (mFISH) or by a digital PCR test (iCS-digital PSC test developped by Stem Genomics to detect recurrent genetic abnormalities).<br>Once published, full characterisations of each iPS cell line will also be available on the hPSCreg global online registry for iPS cell lines (https://hpscreg.eu).
Mycoplasma contamination	Absence of mycoplasma contamination was verified by theMycoAlert™Mycoplasma Detection Kit (selective biochemical test of mycoplasma enzymes) used according to the manufacturer's instructions (Lonza)
Commonly misidentified lines<br>(See ICLAC register)	No commonly misidentified cell lines used.
